# Supplementary material for: Cellular interactions within the immune microenvironment underpins resistance to cell cycle inhibition in breast cancers
Source: Nat Commun. 2025 Mar 3;16:2132. doi: 10.1038/s41467-025-56279-x (PMC11876604; doi:10.1038/s41467-025-56279-x)
Supplement: Supplementary file 1 — Supplementary Information [file 41467_2025_56279_MOESM1_ESM.pdf]

Supplementary information for: Cellular interactions within the immune microenvironment underpins resistance to cell cycle inhibition in breast cancers

Authors:

Jason I. Griffiths[1,2,*, †], Patrick A. Cosgrove[1, †], Eric F. Medina[1], Aritro Nath[1], Jinfeng Chen[1], Frederick R. Adler[2,4], Jeffrey T. Chang[5], Qamar J. Khan[3,], Andrea H. Bild[1,*]

Affiliations:
1. Department of Medical Oncology & Therapeutics Research, City of Hope National Medical Center, 1500 East Duarte Road, Duarte, CA, 91010, USA.
2. Department of Mathematics, University of Utah 155 South 1400 East, Salt Lake City, UT, 84112, USA.
3. Division of Medical Oncology, Department of Internal Medicine, The University of Kansas Medical Center, Kansas City, KS, 66160, USA.
4. School of Biological Sciences, University of Utah 257 South 1400 East, Salt Lake City, UT, 84112, USA.
5. Department of Integrative Biology and Pharmacology, School of Medicine, School of Biomedical Informatics, UT Health Sciences Center at Houston, Houston, TX, 77030, USA.

* Corresponding authors: Jason Griffiths (jasonigriff@gmail.com), Andrea Bild (abild@coh.org)

† Equally Contributing Authors.

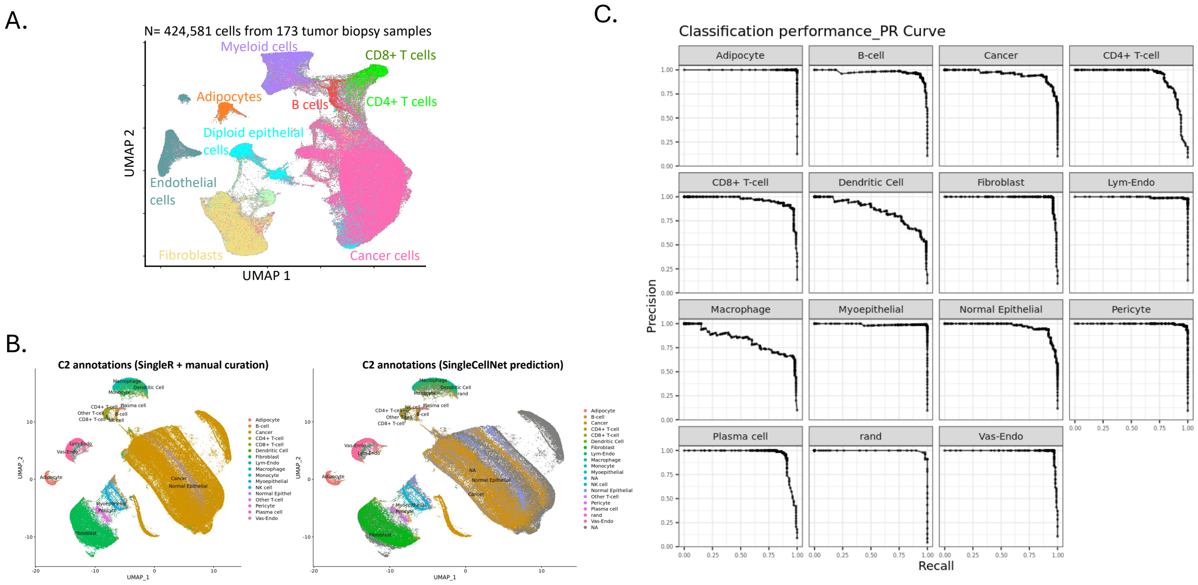

Figure S1) Classification of phenotypically diverse cancer and non-cancer cells within the tumor microenvironments of early-stage ER+ breast cancer patient tumors during cell cycle or endocrine therapy. A) A total of 424,581 high quality cells were transcriptionally profiled from 173 serial biopsy samples of 62 patient tumors from the discovery and validation cohorts. Distinction of cancer and non-cancer cell types shown by UMAP dimension reduction plot of single cell gene expression profiles (points= single cells, color= cell type). B) Validation cohort cell annotations can be predicted using both machine learning of the cell type transcription profiles observed in the discovery cohort or using independent SingleR annotation and manual curation. This UMAP projection shows high-quality validation cohort cells grouped by cell type predicted from their gene expression profiles. The UMAP was calculated from the first 10 principal components of the scaled expression of 100 marker genes associated with each cell type. The left panel shows cells grouped and annotated using SingleR and manual curation while the right panel shows cells grouped and annotated using predictions of SingleCellNet random forest machine learning classifier. C) Precision recall curves of the random forest classifier in the hold-out validation subset of high-quality validation cohort cells.

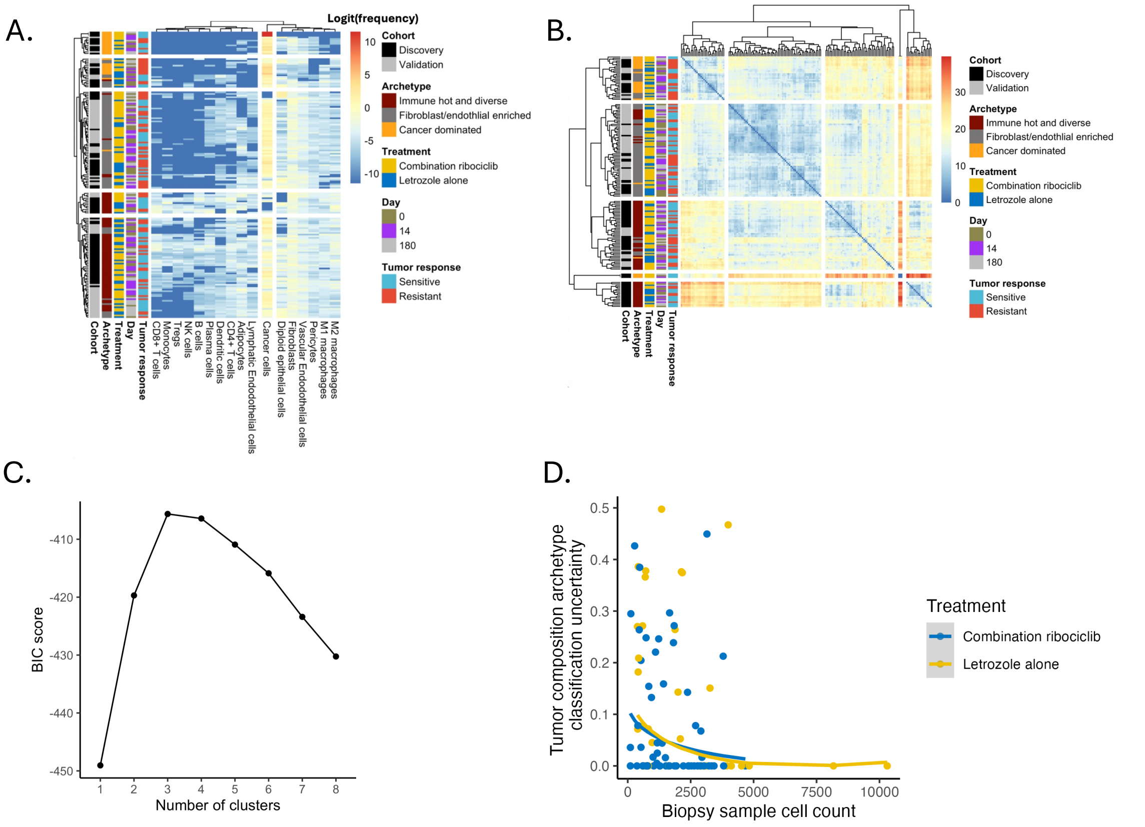

Figure S2) Tumor composition clustering, archetype detection and classification uncertainty analysis. A) Heatmap of the relative frequency of high-quality cells of each cell type within tumor biopsy samples of the discovery and validation cohorts from the FELINE trial. Tumors samples (y-axis) and cell types (x-axis) are clustered using hierarchical clustering of logit transformed cell type frequencies. For each sample, annotations (sidebar) are provided for the cohort, the archetype assigned by the tumor archetype analysis, treatment received, Day of treatment and the tumors response. (Sample size= 422,635/424,581 annotated cells (ensuring equivalent quality between cohorts) from 173 biopsy samples of 62 patient tumors at 3 timepoints). B) Heatmap representing the distance matrix of the logit transformed cell type frequencies between biopsy samples. Coloration indicates the Euclidean distance between sample compositions (blue=highest similarity, red=greatest difference). Samples are clustered by pairwise distances using hierarchical clustering (dendrogram). For each sample, annotations (sidebar) are provided for the cohort, the archetype assigned by the tumor archetype analysis, treatment received, day of treatment and the tumor’s response. C) Bayesian information criterion (BIC) comparison of gaussian mixture models assuming different number of tumor composition archetypes. Higher scores indicate greater probabilistic support for gaussian mixture models with that number of archetypes, given the single cell composition data. D) Tumor composition archetype analysis classification uncertainty. For each tumor biopsy sample (point) the archetype classification uncertainty (misclassification probability) was associated with the samples cell count with the expectation that more thoroughly sampled tumors should be classified with less uncertainty. The trend line (solid line; generalized additive model expectation) for each treatment group (color) supports this prediction and indicates that classification uncertainty of the least sampled biopsies was still typically below 10% and decreased rapidly with sampling effort.

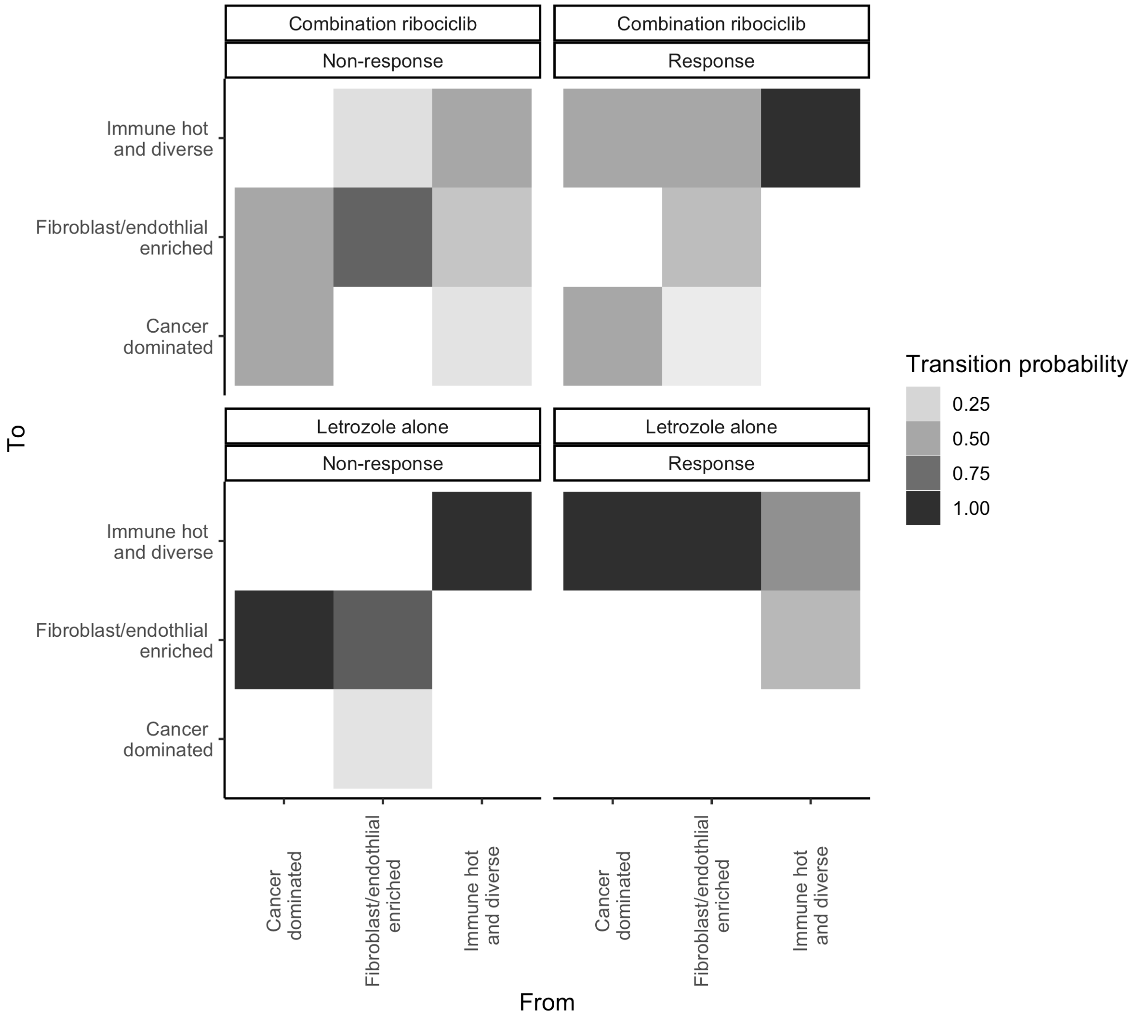

Figure S3) Ribociclib sensitive tumors get hot by the end of treatment independent of the starting state. However, resistant tumors remain in the cancer dominated or fibroblast/endothelial enriched states. Heatmap showing the probability of resistant and sensitive tumors of the discovery cohort receiving combination ribociclib or letrozole alone transitioning between each archetypal tumor composition state. Coloration indicates the proportion of tumors transitioning from one state (x axis) to another (y axis).

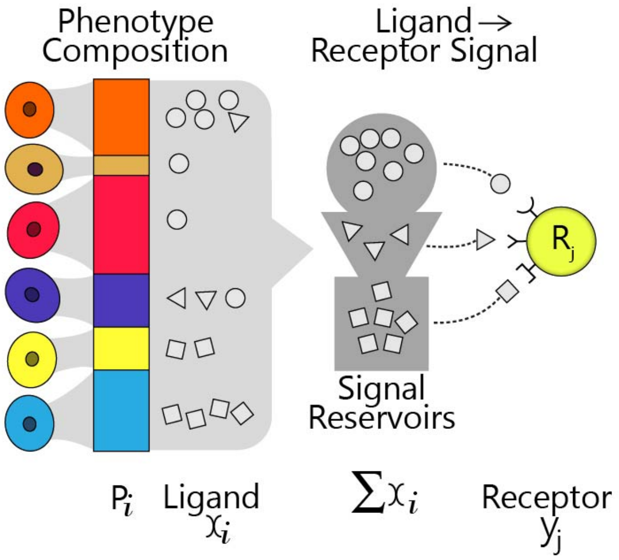

Figure S4) Schematic overview of the extended expression product method used to measure networks of communication between the phenotypically diverse populations of cancer and non-cancer cell types constituting the tumor, accounting for both composition and phenotype. Phenotypically diverse subpopulations of each cell type were resolved using UMAP and relative abundance calculated in each tumor sample (proportion subpopulations i= Pi). For each subpopulation (cell color), ligand expression (shapes) quantifies the per cell signal production and the total across the subpopulation of cell indicates the contribution to signals in the TME. For each signal receiving cell, the corresponding receptor expression quantifies the ability of each single cell to receive signals from the TME. Ligand-receptor communications were defined using a curated LR communication database of known protein-protein ligand-receptor interactions (99). This defined a set of 1444 LR communication pairs through which cells may communicate.

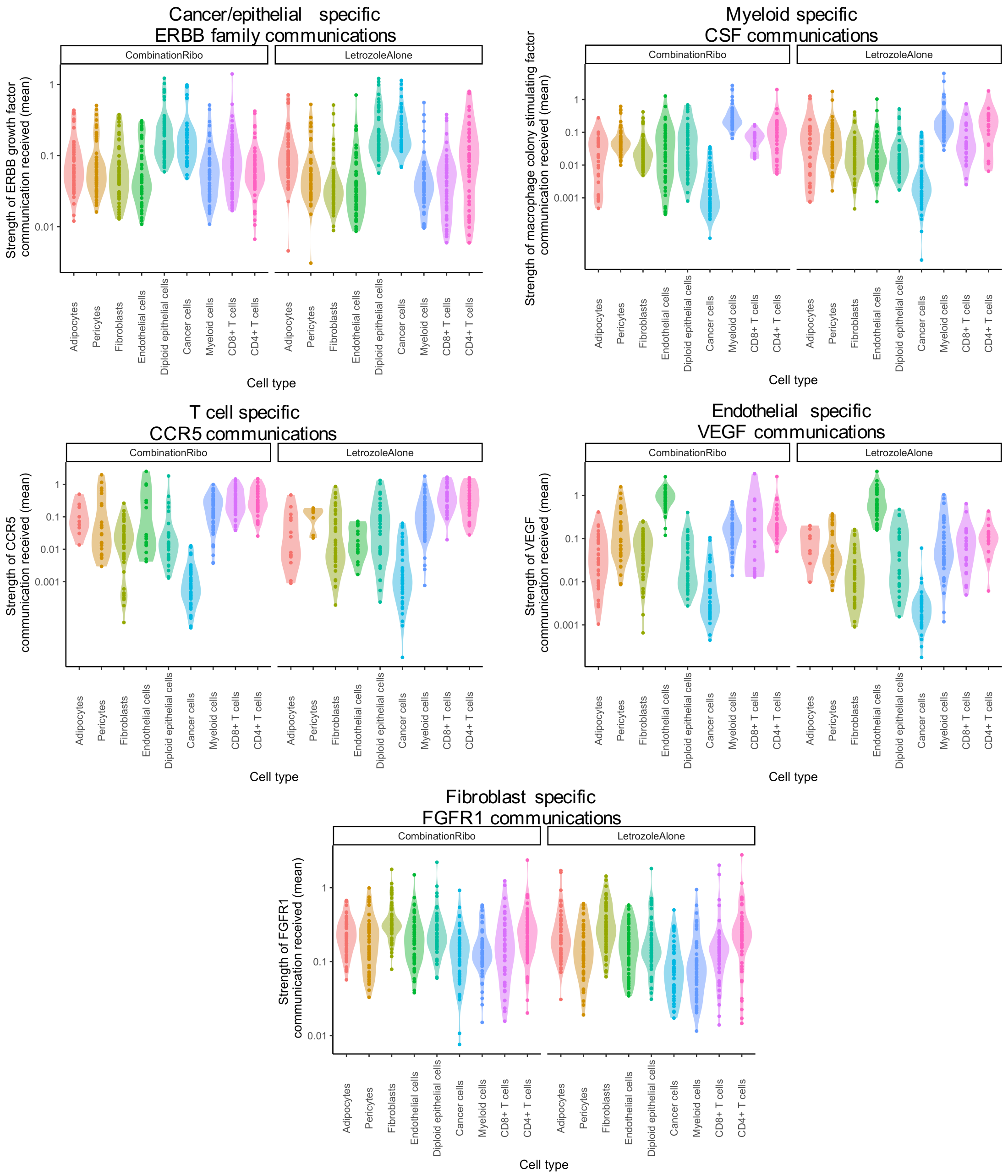

Figure S5) Validation that the extended expression product method recovers known communications specifically received by a certain cell type and providing essential signals for activation (e.g., VEGF is an angiogenic factor and essential growth factor for vascular endothelial cells). Violin plots show the log scale strength of communication (y axis) received by cells of each cell type (x axis: color) through 5 cell-type specific communications. As expected, epithelial (normal and cancer) cells received higher communications via ERBB family receptors (EGF+ERBB2-4), macrophages received higher communications via macrophage colony-stimulating factor 1 receptor (CSF1R), T cells received higher communications via C-C chemokine receptor type 5 (CCR5), endothelial cells received higher communications via vascular endothelial growth factor (VEGF) and fibroblasts received higher communications via fibroblast growth factor receptor 1 (FGFR1). Each cell type specific communication was found to be significantly activated in the expected cell type (all $p<0.005$).

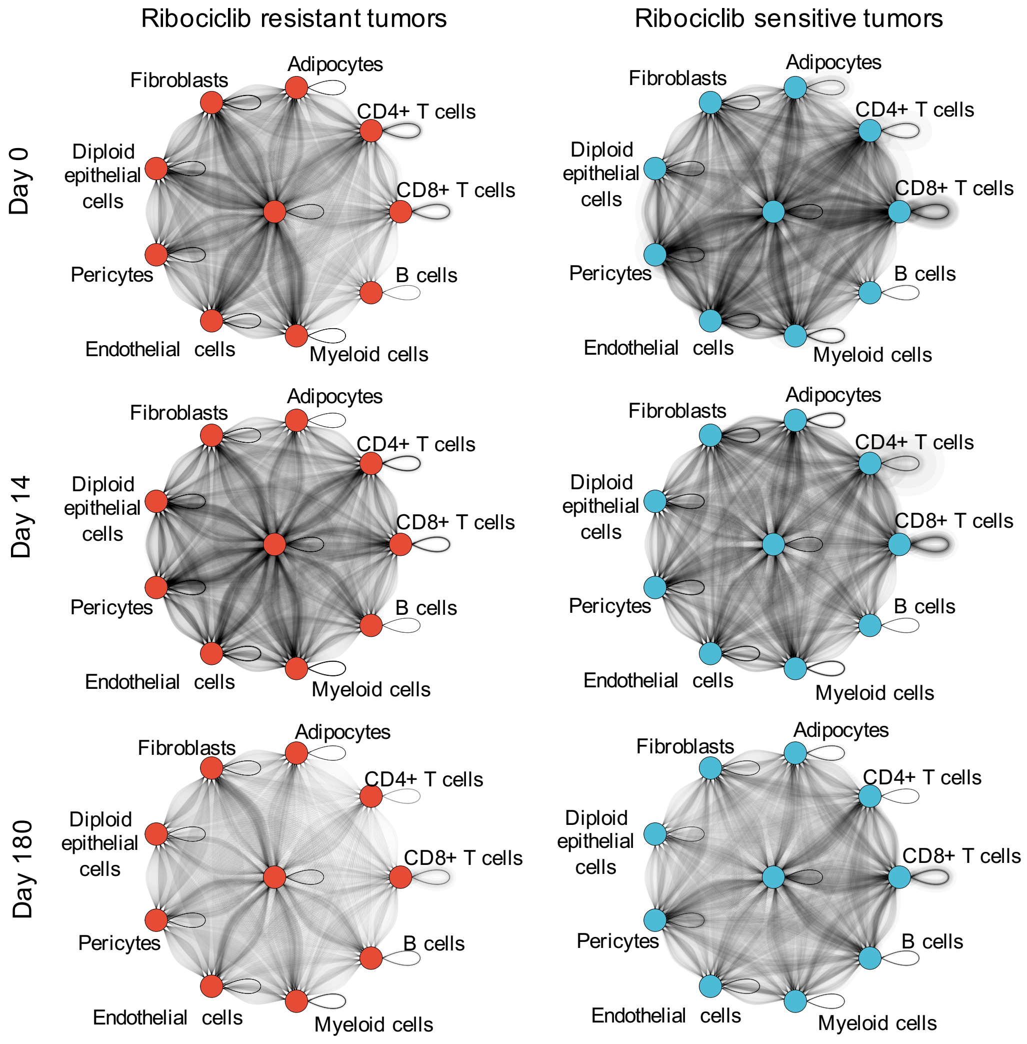

Figure S6) Global communication differences between ribociclib resistant and sensitive tumors. Measurements of tumor wide communication between cell type populations before (top row) during (middle row) and after treatment (bottom row) in ribociclib resistant (left column) and sensitive (right column) tumors of the discovery cohort. Communication networks show cell types (nodes) and their strength of communication with each other via each LR communication pathway (edges). The darkness of the edge lines is proportional to the strength of communication via that pathway. All edges are directed from one cell type to another, going in a clockwise direction from the sender and to the receiver.

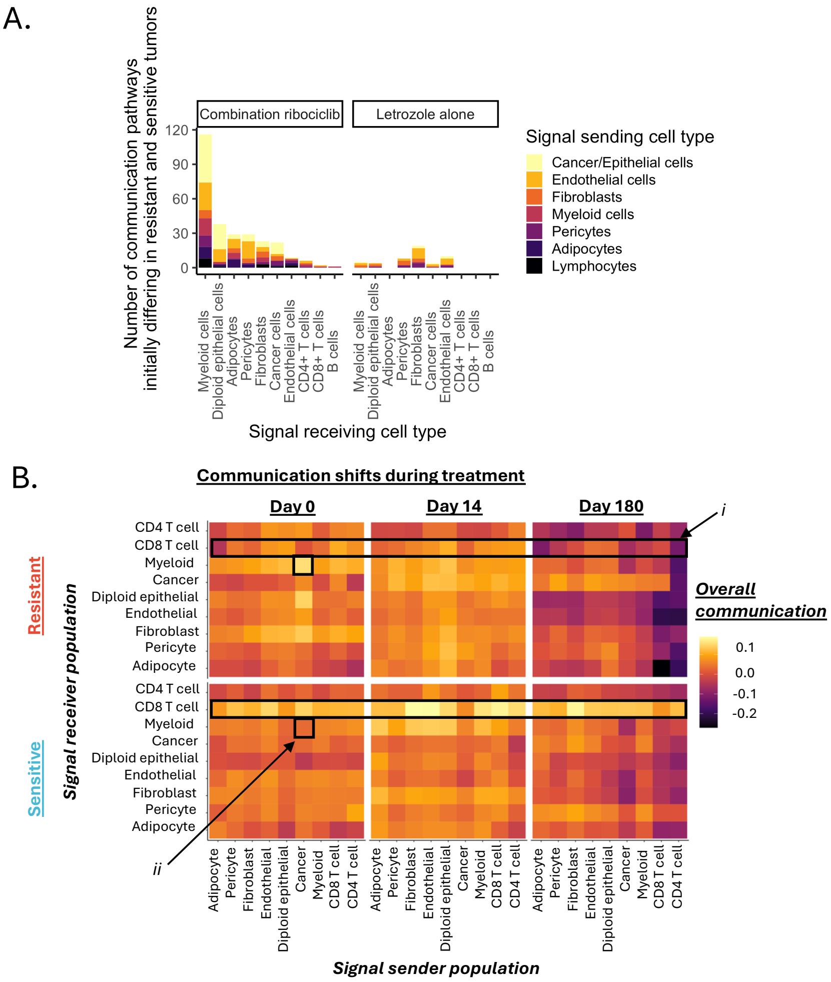

Figure S7) Pre-treatment and dynamic differences in communication within in tumors sensitive and resistant to cell cycle inhibitor therapy. A) Frequent communication differences between cell types in tumors resistant and sensitive to treatment. The y-axis indicates the number of pre-treatment communications that each cell type receives from each other cell type which differed between tumors resistant and sensitive to each treatment (panels). Colors indicate which cell type is sending the signals. The left panel shows the number of pre-treatment dysregulated communications from cancer/epithelial cells to myeloid cells in resistant tumors of the discovery cohort (all communication pathways detected by ANOVA with adjusted p-values <0.05). The right panel shows the contrasting differences in communication seen between letrozole resistant and sensitive tumors of the discovery cohort, with fibroblasts being the target of most dysregulated communications. B) Summarization of communication measurements reveal changes in the overall communication networks in tumors sensitive and resistant to cell cycle inhibitor therapy (rows) before during and after treatment (columns). Heatmap shows the pairwise communication between cell types during treatment. Overall communication measures the average signaling between one cell type and another across all L-R communication pathways. Coloration indicates the strength of communication (coloration) from one cell type (x axis) to individual cells of each other cell type (y axis) in the discovery cohort. Boxes indicate: i) the loss of communication with CD8+ T cells during treatment in ribociclib resistant tumors, ii) the stronger communication of cancer cells with myeloid cells prior to treatment in ribociclib resistant tumors.

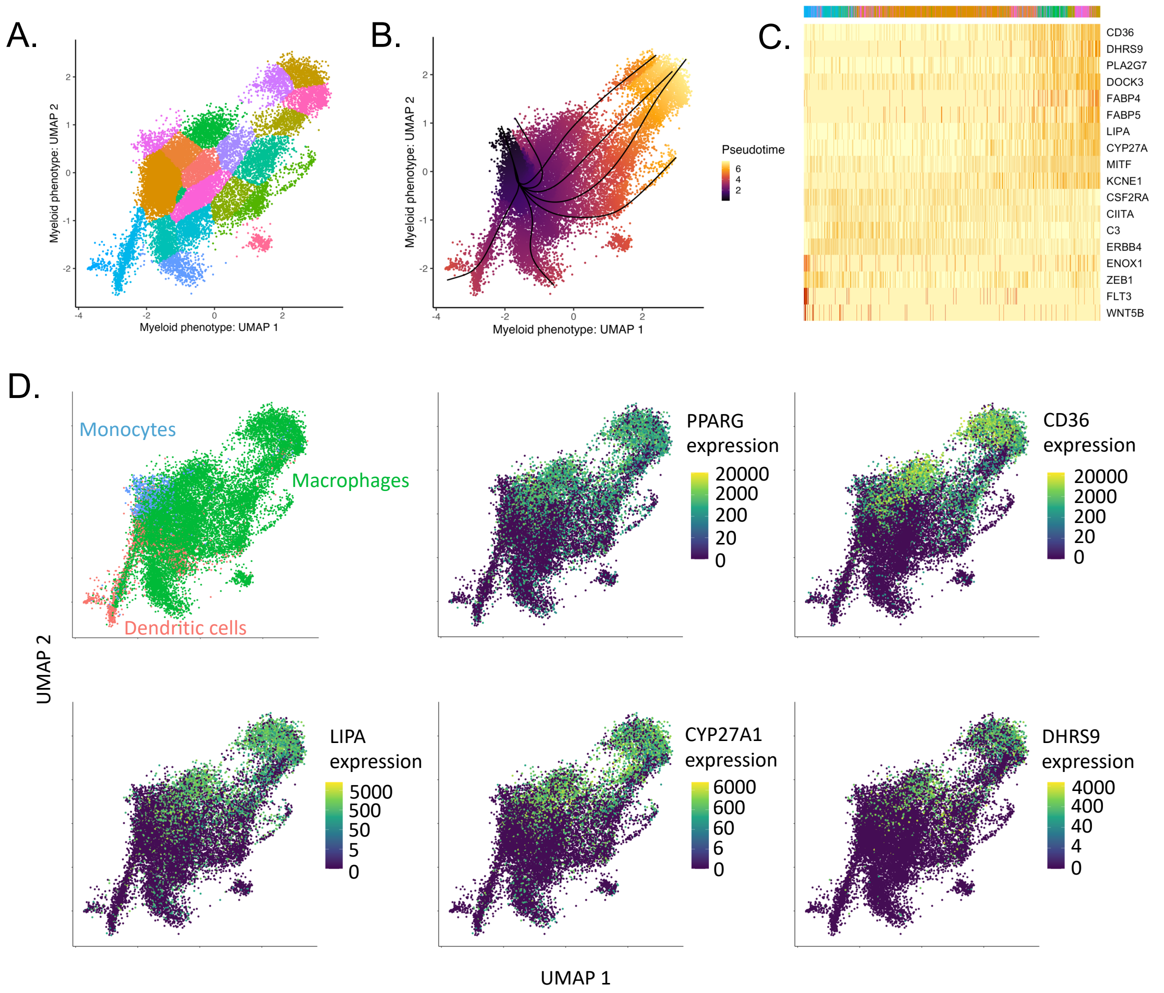

Figure S8) Single cell myeloid phenotype clustering, differentiation trajectory inference and characterization of dynamic changes in gene expression. A) Visualization of myeloid phenotypic heterogeneity characterized by UMAP dimension reduction of cells in the discovery cohort and validation cohort cells projected into the same space. Cells with similar gene expression profiles are located closer together. Myeloid subpopulations with distinct UMAP phenotypes identified using a Gaussian mixture model, with Bayesian Information Criteria (BIC) used to determine the number of phenotype clusters (n= 21). Cells are colored by cluster. Sample size: n=27127 myeloid cells from 167 of the 173 biopsies from 61/62 patient tumors (Discovery: 10940 cells, Validation: 16187 cells). B) Single cell myeloid differentiation trajectories inferred, using the slingshot algorithm to construct a minimum spanning tree (MST) between UMAP coordinates of myeloid phenotype clusters, revealing the number and trajectory of differentiation lineages. The MST was constrained based on the known biology that monocytes progenitors differentiate into macrophage cells. Simultaneous principal curves provided smooth representations of the differentiation trajectories of various lineages (black lines show the 7 trajectories detected). Pseudotime values of each cell (point coloration) were obtained by orthogonal projection onto the curves. This average pseudotime value measures the cell’s distance along the MST from the root node (monocyte state). C) Core transcriptional program defining M1-/M2-like myeloid polarization. Heatmap shows the single cell expression of 18 genes (rows) that have a known association with M1/M2 differentiation and showed increasing or decreasing

expression with polarization along pseudotime trajectories. Columns represent single myeloid cells that are ordered by their polarization pseudotime value (annotation bar above indicates myeloid subpopulation cluster annotation). Genes dynamically expressed across polarization pseudotime were identified using a general additive model to test the potentially nonlinear relationships between gene expression and polarization pseudotime. M1/M2 differentiation association genes were identified from the 40 dynamically expressed genes that increased and decreased in expression with pseudotime. D) Identification of macrophages with an immune-suppressive M2-like polarized phenotype. Analysis of the correlation of gene expression with each axis of macrophage phenotypic variation within macrophage cells showed that the second UMAP dimension characterized the gradient of differentiation from an M1-like immune-activating phenotype (bottom) to an M2 pro-tumor immune-suppressive phenotype (top). The top ten genes most correlated with the UMAP 2 phenotype included the following five M2-like phenotype marker gene: CD36, CYP27A1, DHRS9, LIPA, PPARG. Heatmaps show the gradient of expression of each of these immune-suppressive M2 marker genes across that macrophage phenotype landscape (points=single cells; color gradient= CPM expression).

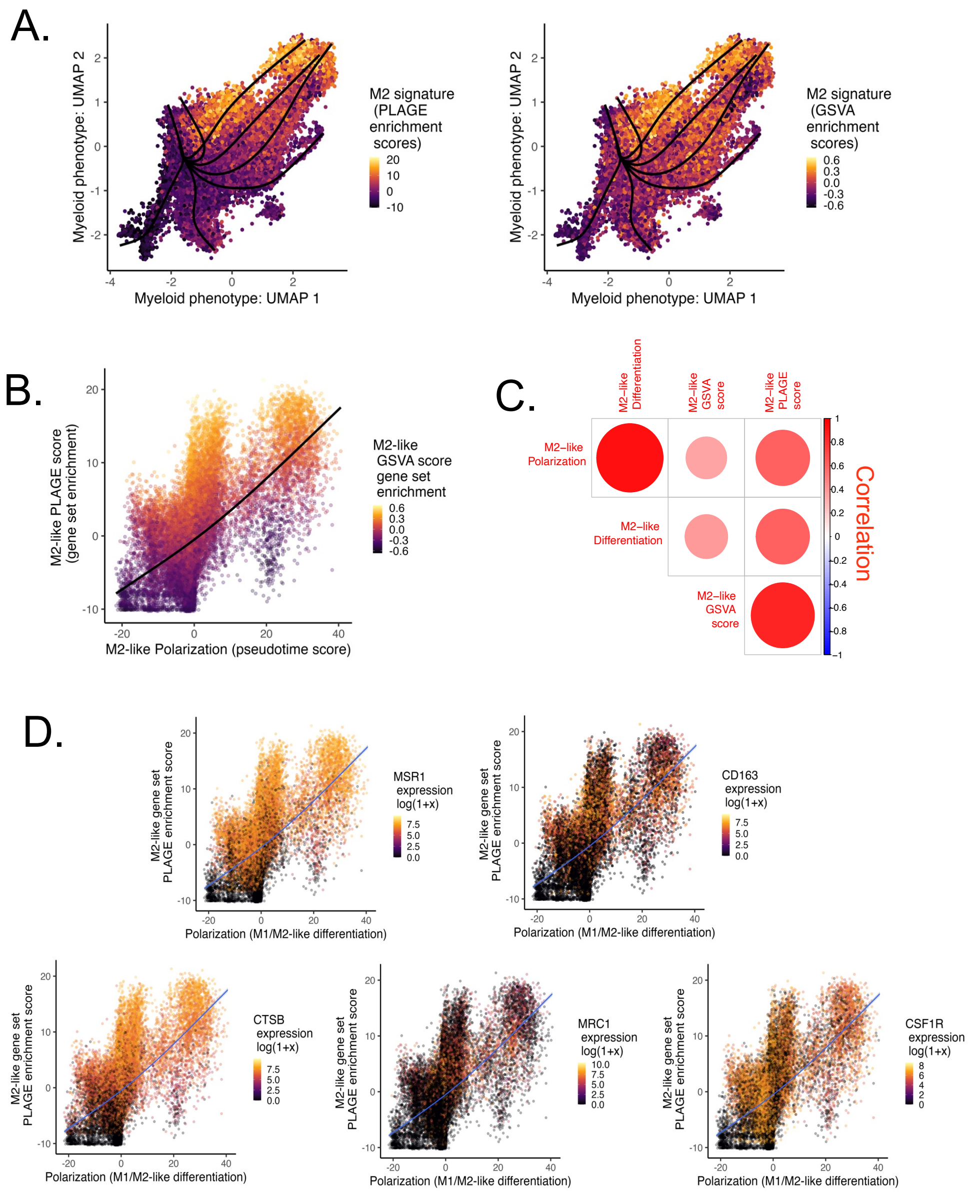

Figure S9) Data-driven analyses (UMAP and pseudotime reconstruction) and knowledge-based characterization of myeloid phenotypes (M2 macrophage gene signatures enrichment and marker gene assessment) provide an integrated and highly consistent characterization of myeloid differentiation from a monocyte progenitor and polarization to either an M1- or M2-like state.
A) Scatter plots show enrichment (coloration) of the knowledge-defined M2-like differentiation gene set signature, derived from pan cancer studies of cancer-associated myeloid cells (n=36 genes; Supplementary Data 30), in myeloid cells (points) with polarization along pseudotime trajectories (curves) towards high UMAP 2 differentiation scores (y-axis). Consistent results were obtained when characterizing M2-like differentiation by their expression of established marker genes using Pathway level analysis of gene expression (PLAGE; singular value decomposition) (left-side) or Gene set variation analysis (GSVA) (right side).
B) Agreement between myeloid polarization (x-axis) from an M1-like state (negative values) to an M2-like state (positive values), measured by pseudotime reconstruction, and the two approaches to quantify M2-like gene set enrichment

scores using PLAGE (y-axis) or GSVA (color). Points represent single myeloid cells from the discovery and validation cohorts of the FELINE trial. The non-linear association between gene set enrichment and pseudotime polarization (black line) was characterized using a generalized additive model (PLAGE trend: edf=1.99, F=8273, p=2e-16; GSVA trend: edf=2.00, F=2015, p=2e-16). C) Correlation plot showing strong positive correlation (color) and consistency between all metrics to quantify M2-like myeloid differentiation. Circle color indicates the Pearson correlation coefficient between pairs of metrics (red= positive, blue= negative, grey=no correlation) and the size of the circle shows the absolute value of the coefficient (coef:: mean=0.62, range=0.35-0.93). D) Increased myeloid polarization is linked to higher gene expression (color) across a set of established M2 myeloid cell markers. Scatted plots show the association between myeloid cell polarization (from pseudotime), and their M2-like macrophage gene set signature (PLAGE enrichment score). In each panel, single myeloid cells (points) are colored by their gene expression of canonical M2 markers. As in panel B, the non-linear association between myeloid polarization and M2-like PLAGE signature scores are summarized by a generalized additive model (solid line; shaded regions= 95% confidence interval). Sample size= 27127 myeloid cells from 62 patients.

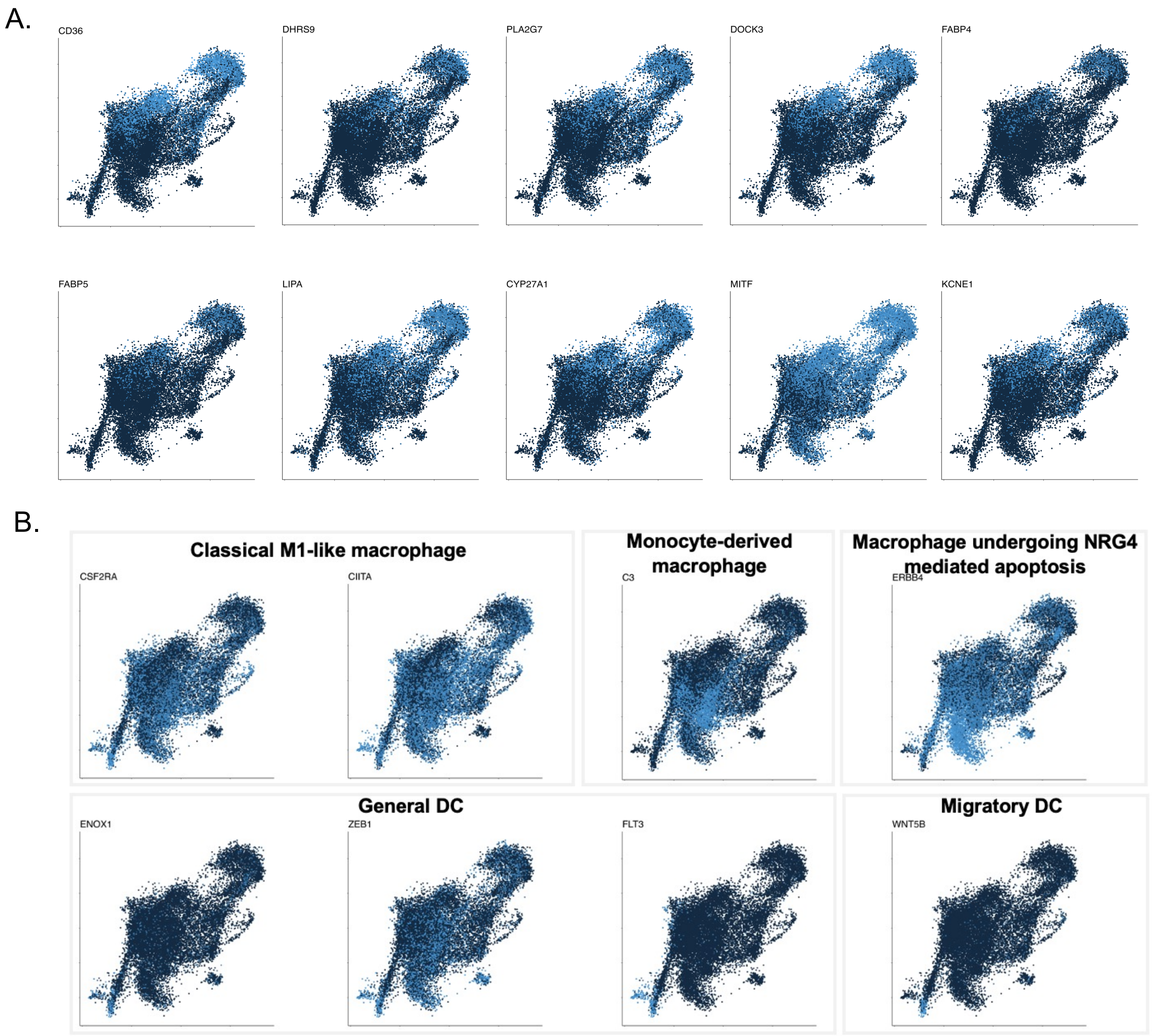

Figure S10) Heterogeneity in myeloid subpopulation biomarker gene expression across the myeloid UMAP phenotypic landscape. A) M2 macrophage associated marker gene expression (color) is overlaid onto the UMAP dimension reduction coordinates of single cells from the discovery and validation cohorts (Sample size: Discovery: 10940 cells, Validation: 16187 cells). B) M1/DC macrophage marker genes expression is overlaid onto the UMAP dimension reduction coordinates of single cells from the discovery and validation cohorts (Sample size: Discovery: 10940 cells, Validation: 16187 cells). Points indicate single cells (color gradient= scales(log(1+CPM)) expression). Cells with similar gene expression profiles are located closer together.

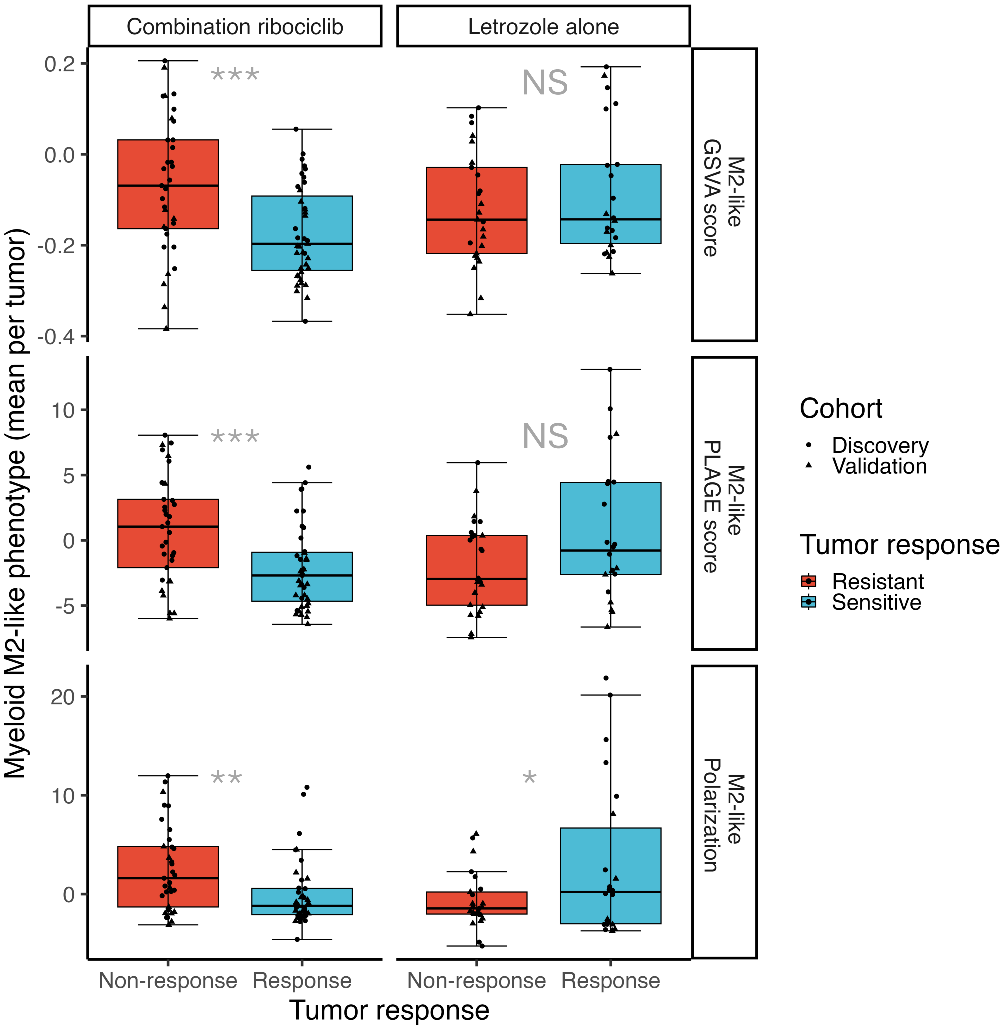

Figure S11) M2-like macrophage differentiation early in treatment in growing ribociclib-resistant tumors confirmed using various knowledge- and data-based approached to measure myeloid phenotypes. Box and whisker plot showing the increased average M2-like phenotype of myeloid cells in resistant tumors (red) compared to sensitive tumors (blue) early (Day 0-14) in combination ribociclib treatment (left panels) but not letrozole alone (right panels). Consistent results were obtained when characterizing M2-like differentiation by their expression of established marker genes using either Gene set variation analysis (GSVA) or Pathway level analysis of gene expression (PLAGE; singular value decomposition) as well as by characterizing myeloid polarization through pseudotime reconstruction (slingshot applied to UMAP coordinates). Linear model comparisons of each M2-like phenotype metric (rows) made between resistant and sensitive tumors under combination ribociclib (GSVA Est=0.11, sd=0.030, t=3.51, p=0.00078; PLAGE Est=3.05, sd=0.86, t=3.56, p=0.00067; Polarization Est=2.62, sd=0.91, t=2.88, p=0.0051) and letrozole alone treatment (GSVA Est=-0.035, sd=0.039, t=-0.88, p=0.38; PLAGE Est=-2.56, sd=1.32, t=-1.93, p=0.059; Polarization Est=-3.69, sd=1.72, t=-2.15, p=0.037). Sample size: 61 patient tumors (Treatment: 37 combination ribociclib, 24 letrozole alone).

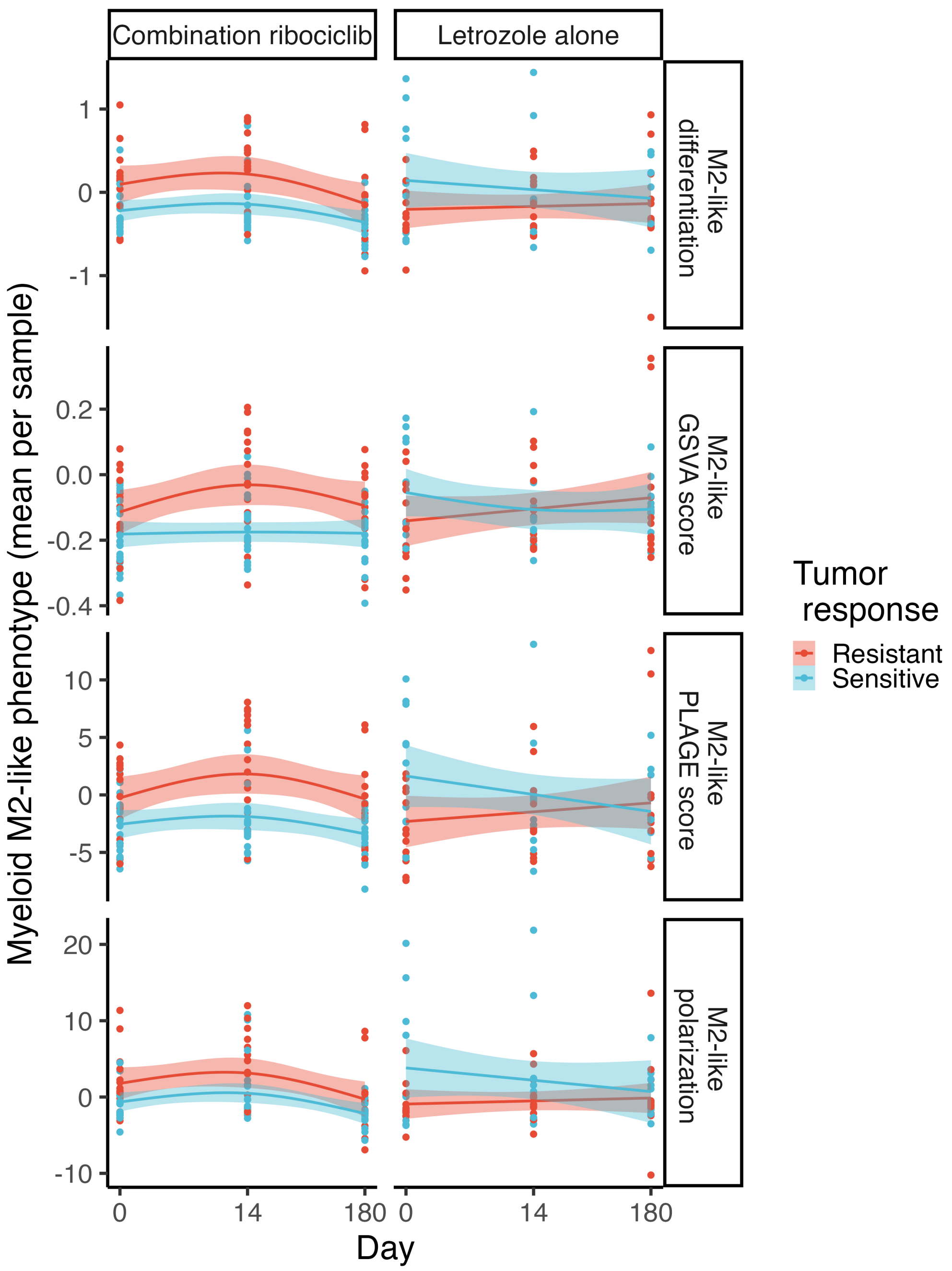

Figure S12) Differences in average myeloid phenotype before and during treatment (columns), in tumors resistant (growing=red) or sensitive (shrinking=blue) to therapy. Pathway trends across tumors were determined using a generalized additive model (solid lines). Confidence intervals of model estimates are shown by shaded regions. The mean M2-like phenotype of each tumor sample was measured using four different approaches (rows). The M2-like differentiation was inferred by applying UMAP dimension reduction to myeloid transcriptomic profiles. In contrast, the M2-like GSVA and M2-like PLAGE scores were calculated by measuring the gene set enrichment (GSE) of established myeloid markers using either Gene set variation analysis (GSVA) or Pathway level analysis of gene expression (PLAGE). Finally, M2-like polarization was measured through pseudotime reconstruction (slingshot applied to UMAP coordinates).

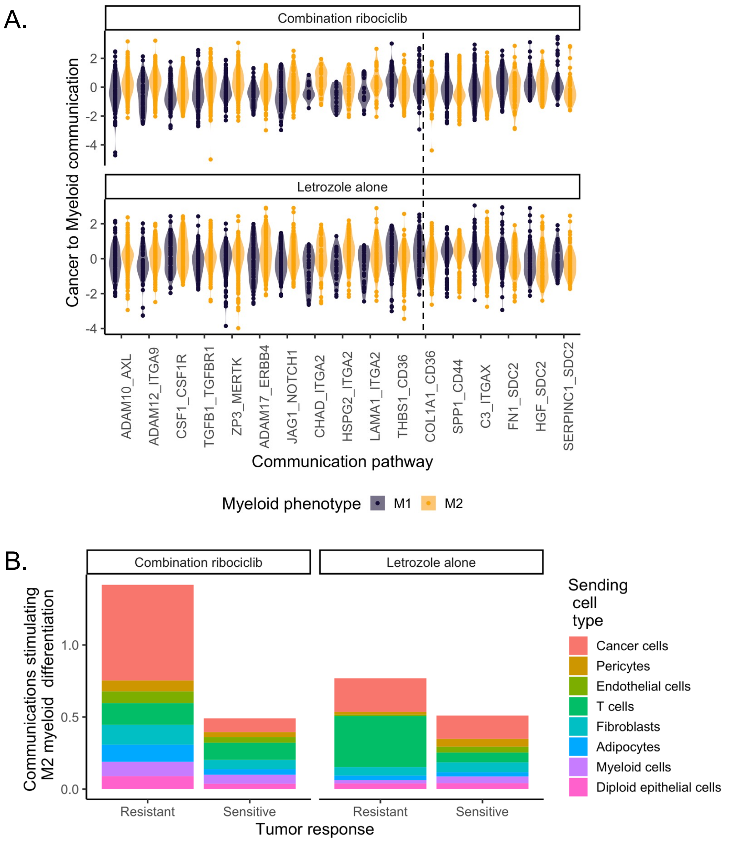

Figure S13) Known M2 differentiation signals were sent by cancer cells and targeted M2-like macrophages. A) Violin plot of established M2 differentiation signals, including CSF1-CSF1R, ADAM10-AXL and ZP3-MERTK, that were sent by cancer cells and differentially targeted to M2 macrophages. Cancer communications targeting M2-like macrophages were identified by analyzing the average strength of communications from individual cancer cells to macrophages differentiated to either an M1- or M2-like phenotype (above vs below average differentiation). The LR communications that cancer cells used to differentially communicate with each macrophage phenotype were detected using linear models. Across treatments, cancer cells communicated more strongly with M2-like macrophages using communication pathways on the left-hand side of the dashed vertical line and communicated more with M1-like macrophages using pathways on the right-hand side (all differences in communication significant across treatments with $p<0.005$). B) Cancer cells are the primary contributors of the pre-treatment (Day 0) M2 macrophage differentiation communications promoting the myeloid phenotype shift seen in ribociclib resistant tumors. For tumors resistant and sensitive to each treatment, we calculated the average pre-treatment contribution of each cancer and non-cancer cell type to the M2 differentiation communications received by macrophages (y axis) in resistant versus sensitive tumors (x-axis), totaling the signals across scaled LR pathways. Cancer cells were found to be the dominant contributors to M2 differentiation communications in ribociclib resistant tumors (left panel). The tumor-wide strength of communication (total bar height) was stronger in resistant tumors, with the cancer cell contribution (left-side cancer section) surpassing that of all cell types in the sensitive tumors (total bar height). This suggests that M2-like macrophage differentiation in ribociclib resistant tumors was driven primarily by corrupting cancer communications that evolved prior to treatment.

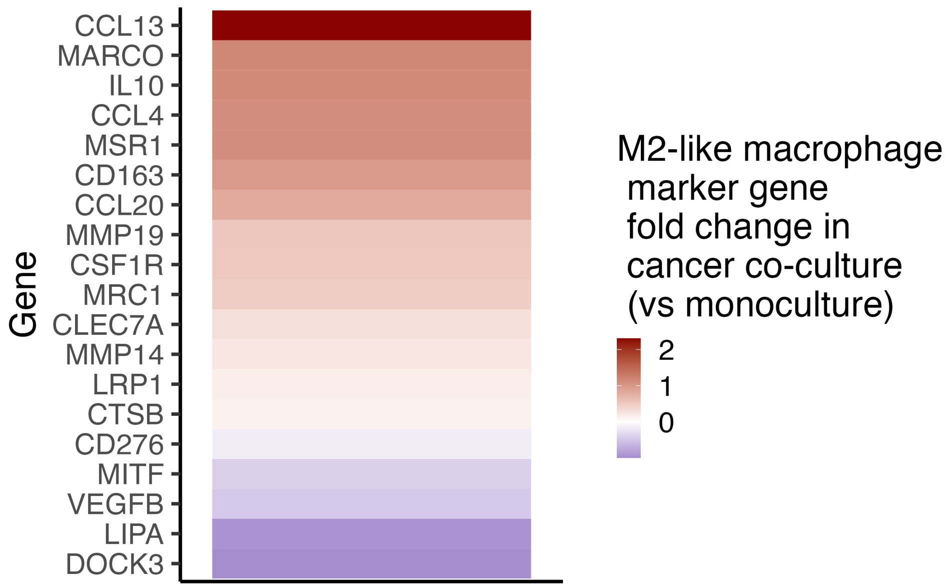

Figure S14) Monocyte cells increase expression of established M2-like marker genes when cocultured with breast cancer cells, compared to monocyte monocultures grown in isolation. Molecular data was generated by Hollmén et al. 2015, following a transwell experimental which isolated the effects of cancer-myeloid communication on myeloid phenotype from effects of direct contact. They performed whole transcriptome sequencing of mono-/co-cultured myeloid cells. We used differential expression analysis to compare the expression of known M2-like macrophage marker genes (Supplementary Data 30) between culture conditions. Heatmap shows the fold change in expression of 19 differentially expressed M2-like macrophage marker genes when monocytes were grown in cancer co-cultures. Most differentially expressed M2-like macrophage markers (rows) were increased in cancer co-cultures (red; 14/19 marker genes), whilst only a few decreased (blue).

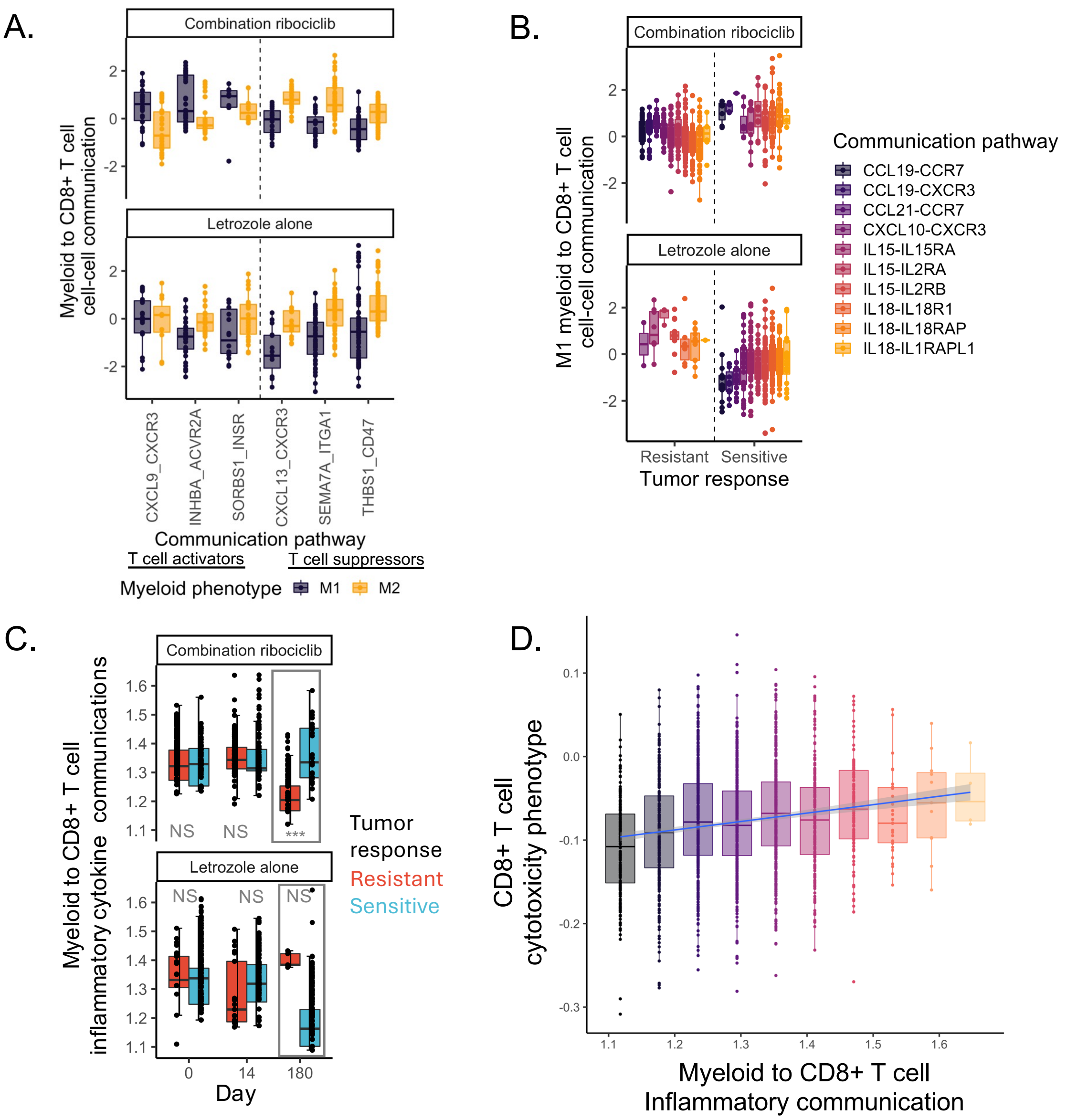

Figure S15) Suppression of immune activating signals from M1-like myeloid cells to CD8+ T cells by end of treatment in ribociclib resistant but not sensitive tumors is linked to CD8+ T cell activation to an effector phenotype. A) Verification that M1-like macrophages send primarily immune activating signals whereas M2-like macrophages send more immune suppressing signals to CD8+ T cells. Boxplots show the differential communication of individual M1- and M2-like myeloid cells with CD8+ T cells under each treatment (rows). The M1- and M2-like macrophage specific communications with CD8+ T cells were identified by comparing individual cell-cell communication, via different LR pathways across tumors. Hierarchical random effects models detected communications differentially sent by individual M1- or M2-like macrophages to CD8 T cells, while controlling for the activation state of the signal receiving T cell. Multiple comparisons were accounted for using FDR correction. As expected, the M1-like macrophages sent stronger immune-activating signals to T cells (left of vertical dashed line) whereas the M2-like cells sent immune-suppressing signals (right of vertical dashed line). Notably, M1-like macrophages amplified immune-activating signals much more in ribociclib treated tumors compared those given letrozole alone. For example, inhibin (INHBA) communications inhibit the development of a regulatory T cell (Treg)

phenotype. This T cell activating communication was more highly activated in tumors receiving ribociclib compared to letrozole alone. Similarly, M1-like macrophages amplified inflammatory communications via C-X-C Motif Chemokine Receptor 3 (CXCR3) in ribociclib tumors more than in tumors given letrozole. These results support the central role of macrophages in activating or suppress an antitumor immune response and driving divergent immune compositions and treatment response of resistant and sensitive tumors. B) Suppression of immune activating signals from individual M1-like macrophages to CD8+ T cells in ribociclib resistant versus sensitive tumors. To measure the ability of individual macrophages to activate an immune response, we next contrasted the M1-like macrophages cell-cell communications with cytotoxic CD8+ T cells between resistant and sensitive tumors (points=per M1-like cell communication with individual CD8+ T cells). Significant differences in cell-cell communication between resistant and sensitive tumors detected using a hierarchical random effects model. In ribociclib resistant tumors (top panel), individual M1-like macrophages sent substantially weaker immune-activating signals to CD8+ T cells compared to those of sensitive tumors, including suppression: CCL19/21 stimulation of T cell expansion ($df=39$, $t=3.68$, $p<0.001$), CXCL10 promotion of effector T cell differentiation ($df=18$, $t=2.28$, $p<0.05$), interleukin 15 (IL-15) stimulation of proliferation and survival ($df=111$, $t=83.3$, $p<0.005$) and interleukin 18 (IL-18) activation of an interferon response ($df=60$, $t=4.71$, $p<0.0001$). The suppression of M1-like immune activating signals was not seen in letrozole resistant tumors (bottom row). These findings reveal how cancer induced polarization of macrophages can suppress an effective immune response by reducing the T cell activating communications sent by individual M1-like macrophages. C) Suppression of inflammatory cytokine communications from the myeloid population to CD8+ T cells by end of treatment in ribociclib resistant but not sensitive tumors. Immune activating inflammatory cytokine communication pathways were identified by their receptors using the gene-ontology database. The overall immune activating myeloid to CD8+ T cells communication was measured by totaling the inflammatory cytokine communications from across the diverse myeloid population. We then analyzed how this immune activation communication diverged during treatment in resistant and sensitive tumors. This analysis revealed that the M2 polarized macrophage populations of resistant tumors provided fewer immune activating signals to CD8+ T cells throughout ribociclib treatment ($df=185$, $t=4.48$, $p<0.0001$). Conversely, myeloid signaling to CD8+ T cells was maintained in ribociclib sensitive tumors (stronger communication vs resistant tumors: $df=778$, $t=6.79$, $p<0.0001$) and the suppression of immune activating CD8+ T cell communications was not seen in the letrozole resistant tumors ($df=113$, $t=-1.83$, $p=0.07$). D) Stronger myeloid communication of inflammatory cytokine signals linked to CD8+ T cell activation to an effector phenotype. Scatterplot shows the strongly significant association between the inflammatory cytokine communications from macrophages to CD8+ T cells and the differentiation of that T cell towards a cytotoxic phenotype. The T cell differentiation of cells in the discovery cohort was measured using a CD8 T cell specific ssGSEA pathway contrasting gene expression of naive and cancer killing effector cells (GSE22886 Naive CD8 T cell vs NK cell up). For each CD8+ T cells (points) differentiation state was linked to the inflammatory cytokine communication it received from across the myeloid population. Across both ribociclib and letrozole alone treatments, greater macrophage to T cell inflammatory cytokine communication was associated with activation of an effector T cell phenotype ($df=786$, $t=4.98$, $p<0.0001$).

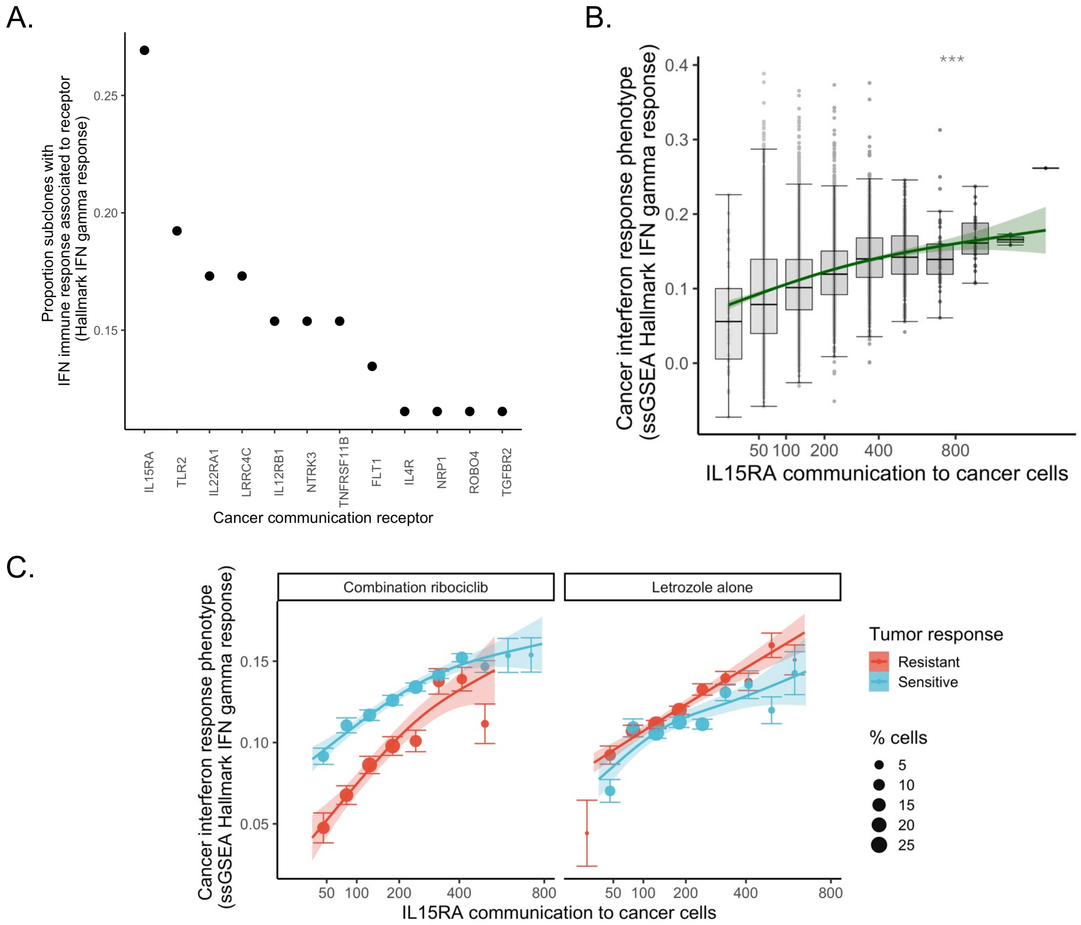

Figure S16) Cancer activation of an interferon gamma immune response post treatment linked to receipt of tumor wide IL15 communications. We assessed the post treatment (day 180) communications most strongly associated with an immune response phenotype in cancer subclones of the discovery cohort. We expected that in immune hot TME's, the high levels of immune activating signals, T cell recruitment and activation should induce a phenotypic response of cancer cells. Cytokine signals should be transduced via JAK/STAT, to the cancer nuclei, driving activation of interferon regulatory factors (IRFs) and induction of IFN-stimulated genes (e.g. Interferon gamma-induced proteins) and increase the production of antigen presenting major histocompatibility complex molecules (MHC I) allowing recognition and killing of cancer cells. The cancer cell activation of this interferon gamma response pathway was measured using the hallmark interferon gamma response ssGSEA scores to contrast pathways activation across cancer cells. For each subclonal cancer population, we identified communication pathways strongly associated with interferon gamma response phenotype activation, using Lasso regression (see methods). Induction of a cancer interferon gamma response was most frequently associated with strong Interleukin 15 Receptor Subunit Alpha (IL15RA) communications from across the TME (28% of tumor subclones) (top left panel). Other detected communications impacting the cancer interferon response bound to cytokine receptors such as: Toll-like receptor 2 (TLR2: 19% subclones), Interleukin 22 Receptor Subunit Alpha 1 (IL22RA1: 17% subclones), Interleukin-12 Receptor Subunit Beta-1 (IL12RB1: 15% subclones). The strongly significant association of IL15RA communication and cancer interferon gamma response was consistent across tumors resistant and sensitive to both treatments (top right panel)

(df=788, t=-2.90, p<0.005). However, cancer cells from ribociclib resistant tumors received weaker IL15RA communications and these cancer cells exhibited the weakest interferon gamma response (bottom panel) (df=788, t=-10.05, p<0.0001). This lack of immune detection was not observed in letrozole resistant tumors. The result was confirmed in the validation cohort, with resistant cancer cells having lower interferon gamma response than those of sensitive tumors under ribociclib but not letrozole treatment (df=26211, t-28.06, p<0.0001).

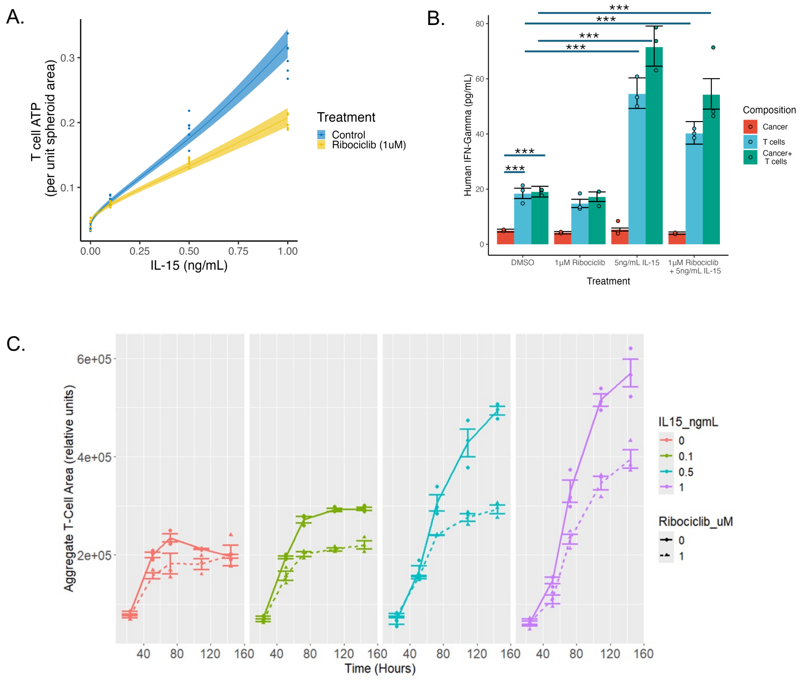

Figure S17) Stimulation of T cell viability, activation and proliferation under IL-15 and combination IL-15 + ribociclib treatment. A) T cell ATP levels increased following IL-15 treatment and overcame the inhibitory effect of ribociclib (observable at low IL-15 doses). Scatterplot showing the impact of IL-15 concentration (x-axis) and ribociclib treatment (color) on T cell ATP levels per unit area of spheroid images (i.e. (total T cell ATP)/(T cell spheroid area)). A linear model describes the log-linear relationship between T cell ATP and square root transformed IL-15 concentrations under ribociclib treated and control conditions (solid line). Shaded regions indicate 95% confidence intervals. IL-15 treatment increased per unit T cell ATP levels (est=2.06, df=44, t=36.57, p=2e-16). Ribociclib opposed this T cell metabolic activation (est=-0.54, df=44, t=-6.74, p=2.76e-8). Sample size: 48 T cell populations; 4 IL-15 levels, 2 ribociclib treatments, 6 replicates. B) ELISA measured IFN-ɣ cytokine production in spheroids containing monocultured cancer cells (CAMA-1), patient derived CD8+ T-cells, or cocultured cancer cells + CD8 T-Cells after treatment with DMSO, 1µM Ribociclib, 5ng/mL IL-15, or 1µM Ribociclib+ 5ng/mL Il-15 for three days (N = 2 samples per treatment). Treatment groups were compared using a two-way ANOVA to test for effects of treatment and composition on log2 IFN-ɣ levels. Ribociclib had no significant effect on cancer or T cell IFN-ɣ production (Cancer: est=-0.074, df=12, t=-0.32, p= 0.75; T cell: est=0.035, df=12, t=0.32, p=0.11). IL-15 increased IFN-ɣ production of T-cells in monoculture and coculture (monoculture: est=1.59, df=12, t=4.91, p= 0.00036; coculture: est=2.07, df=12, t=6.38, p=3.5e-5). Similarly, the combination of IL-15 and ribociclib led to increased IFN-ɣ production of T-cells in monoculture and coculture (monoculture: est=1.17, df=12, t=3.60, p= 0.0037; coculture: est=2.13, df=12, t=6.58, p=2.61e-5). C) T Cell proliferation increased during IL-15 treatment overcoming ribociclib inhibition. T cell proliferation was monitored in monoculture in ULA spheroid plates as used for coculture experiments as measured by total aggregated T cell area over time under. T cells were plated and concurrently treated with IL-15 (0.1, 0.5, or 1ng/mL) and/or 1µM ribociclib treatment 24hrs prior to initial brightfield imaging with Cytation 5 imager. T cell proliferation was monitored every 24 to 48hrs over six days. Sample size: 120 T cell populations; 4 IL-15 levels, 2 ribociclib treatments, 3 replicates.

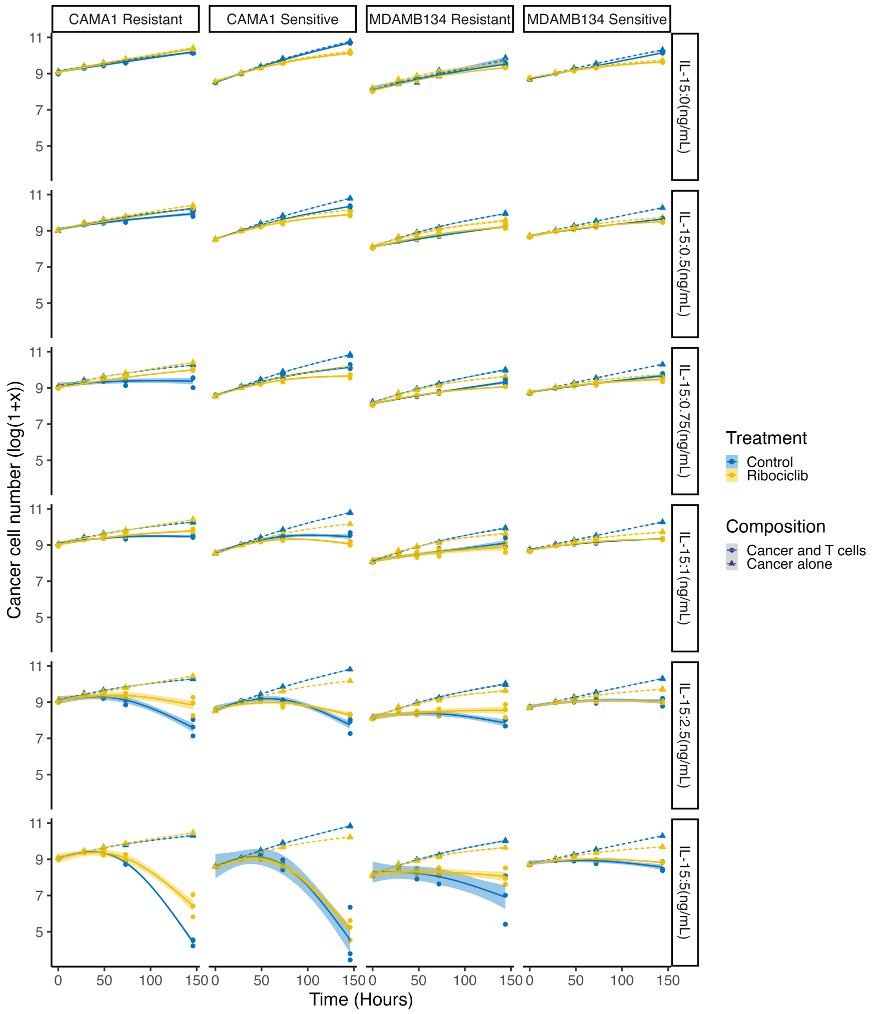

Figure S18) IL-15 enhances T cell control of cancer proliferation in cocultures whereas ribociclib broadly antagonizes T cell control. 3D spheroid growth trajectories of two resistant/sensitive cancer cell line pairs (CAMA-1/MDA-MB-134; left vs right columns) when cocultured with patient derived CD8+ T cells (initially seeded at 4 cancer: 1 T cell ratio) (triangles) or when cancer cells are grown in monoculture (circles). We compared growth trajectories under 6 IL-15 doses (rows; 0-5 ng/mL) and under ribociclib (1uM; yellow) or control (DMSO; blue) treatment. A generalized additive model (GAM) characterized the expected cancer growth curve under each treatment (dashed/solid lines = mono/coculture trajectories). The shaded region indicates the 95% confidence interval of growth, showing times where growth trajectories diverge significantly under ribociclib and control treatments. Without IL-15 addition (top panels), cancer cell growth was poorly controlled by ribociclib treatment and T cells had little effect on cancer spheroid growth. Under low dose IL-15 treatment (middle two rows), T cell activation led to reduced cancer cell proliferation. However, this beneficial effect was lost when ribociclib as added, with the combined effects of coculture and ribociclib treatment being antagonistic. High dose IL-15 T cell activation (bottom row) allowed the control of cancer cell growth with or without ribociclib treatment. Sample size = 288 spheroids, 4 cancer lineages (CAMA-1/MDA-MB-134 resistant/sensitive), 2 compositions (mono/coculture), 6 IL-15 concentrations (0/0.5/0.75/1/2.5/5 ng/mL), 2 ribociclib doses (0/ 1uM), 3 replicates and 5 timepoints.

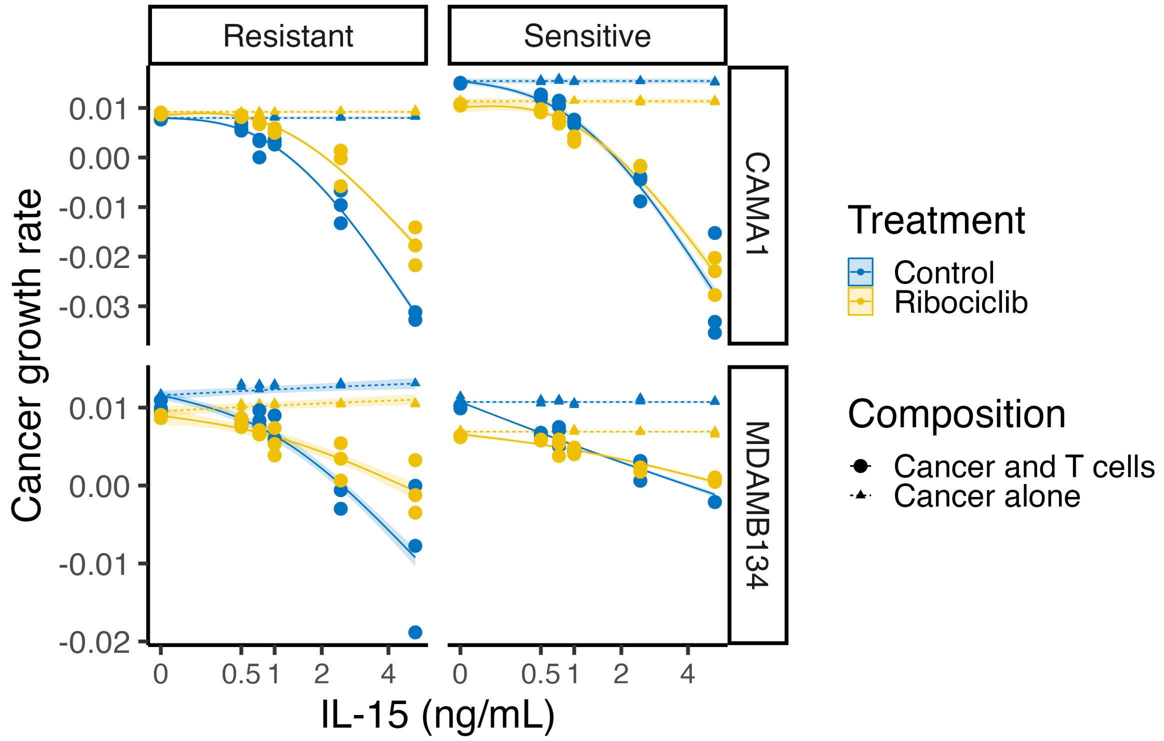

Figure S19) Ribociclib antagonizes T cell control of cancer growth. Impact of IL-15 treatment (x-axis) on the growth rate of 4 cancer lineages (panels: CAMA-1/MDA-MB-134 ribociclib resistant or sensitive) during 6 days of monoculture (triangles) or coculture with patient-derived T cells (circles; 4:1 cancer:T cell ratio), when treated with ribociclib (1uM) (yellow) compared to DMSO control (blue). Points indicate average growth rates of replicate populations, measured by relative growth rate throughout treatment (Sample size = 288 spheroids, 4 cancer lineages (CAMA-1/MDA-MB-134 resistant/sensitive), 2 compositions (mono/coculture), 6 IL-15 concentrations (0/0.5/0.75/1/2.5/5 ng/mL), 2 ribociclib doses (0/ 1uM), 3 replicates). For each cancer lineage, a generalized additive model characterized the expected cancer growth rate under each composition (dashed/solid lines=mono/coculture predictions; shaded region =95% confidence intervals). Cancer growth slowed by IL-15 treatment in T cell coculture, but not monoculture, populations (IL-15 effect in coculutre:CAMA-1 resistant: edf=2.00, F=1323.00,p<1e-16,CAMA-1 sensitive: edf=2.00, F=543.25,p<1e-16,MDA-MB-134 resistant: edf=1.25, F=173.77,p<1e-16,MDA-MB-134 sensitive: edf=1.70, F=684.73,p<1e-16). For all four cell lines, the growth of cocultured cancer cells was less responsive to IL-15 stimulation under ribociclib treatment (yellow circles) than in control cocultures (blue circles) (smaller reduction in cancer growth across the IL-15 gradient; visible by shallower model trend under ribociclib) (ribociclib impact on IL-15 effect in coculutre:CAMA-1 resistant: edf=1.88, F=56.15,p<1e-16,CAMA-1 sensitive: edf=1.00, F=9.61,p=2.7e-5, MDA-MB-134 resistant: edf=1.13, F=6.22,p=5.5e-16,MDA-MB-134 sensitive: edf=1.94, F=51.74,p<e-16). Higher dose IL-15 stimulation was increasingly able to control cancer coculture growth during ribociclib treatment.

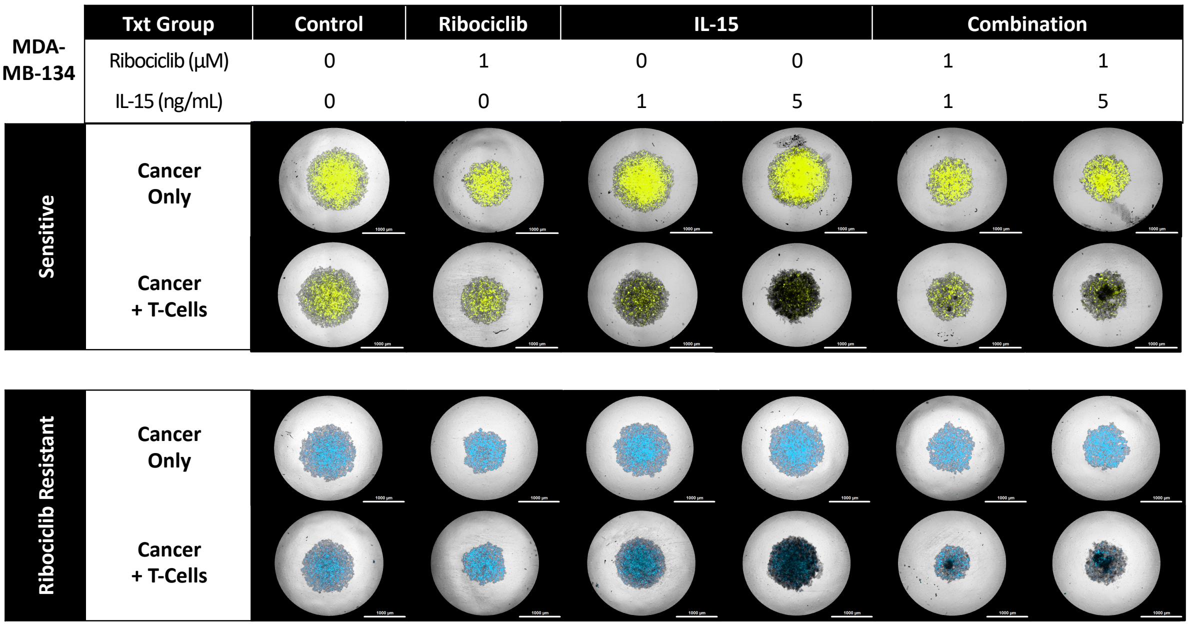

Figure S20) IL-15 T cell activation controls MDA-MB-134 cancer growth. Representative florescent imaging (4X magnification) demonstrating IL-15 activation of an effective cytotoxic T-Cell (unlabeled: black) response in both sensitive (YFP labelled, yellow) or ribociclib resistant (CFP labelled, blue) MDA-MB-134 cancer cells. We compared cancer monoculture (top rows) and cancer-T cell coculture (bottom rows) spheroids following 6 days of treatment with DMSO (0.1%; control), ribociclib (1μM), IL-15 (1, 5ng/mL), or combination ribociclib (1μM) + IL-15 (1, 5ng/mL).

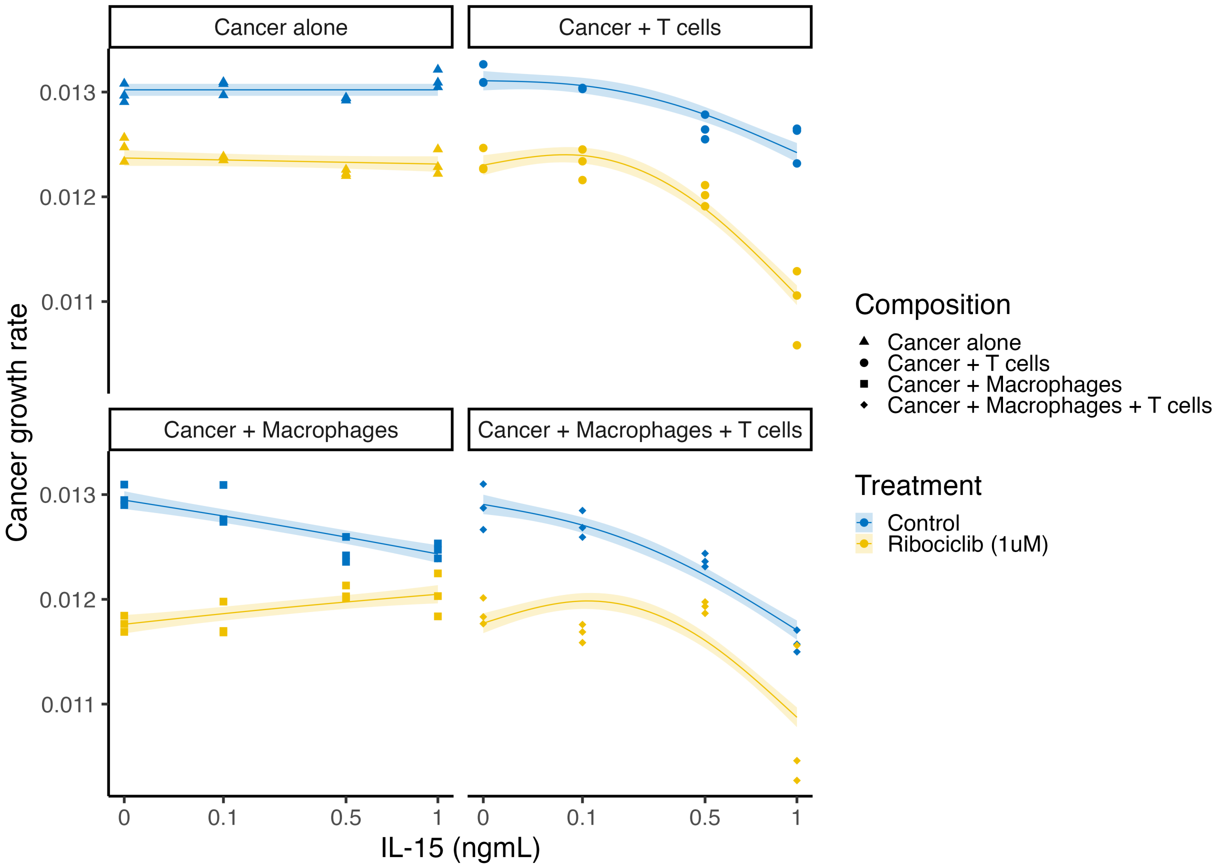

Figure S21) Impact of IL-15 concentration (x-axis) and ribociclib treatment (color) on the growth rate of cancer cells (CAMA-1 ribociclib sensitive) when cultured alone, or with either patient-derived CD8+ T cells, myeloid cells (THP1-M0-like macrophages) or both (panels and shaped indicate spheroid composition). Cancer population growth rates of replicate spheroids were measured over 12 days (points). Sample size = 96 spheroids, 4 cell type compositions, 4 IL-15 concentrations (0/0.1/0.5/1 ng/mL), 2 ribociclib doses (0/ 1uM), 3 replicates. A generalized additive model characterized the expected cancer growth rate under each composition and treatment (solid lines=predictions; shaded region =95% confidence intervals). IL-15 slowed cancer growth in T cell coculture, but not monoculture, populations (IL-15 effect in T cell cocultures without macrophages: edf=1.92, F=18.14, p=2.62e-7). IL-15 also slowed cancer growth in cancer-macrophage cocultures (edf=1.27, F=9.50, p=2.46e-5). Ribociclib decreased the growth rate of cancer populations across coculture conditions (est=-6.51, t=-6.92, p= 9.62e-10). The combination of ribociclib and higher dose IL-15 enhanced the control of cancer growth in both cancer-T cell and cancer-macrophage-T cell cocultures (edf=1.89, F=7.80, p=0.00035). Greater IL-15 concentrations increasingly control cancer growth in T cell or T cell and macrophage cocultures, overcoming the ribociclib immune suppression effects observed at low IL-15 concentrations (indicated by narrowing of gap between ribociclib and control treated cocultures).

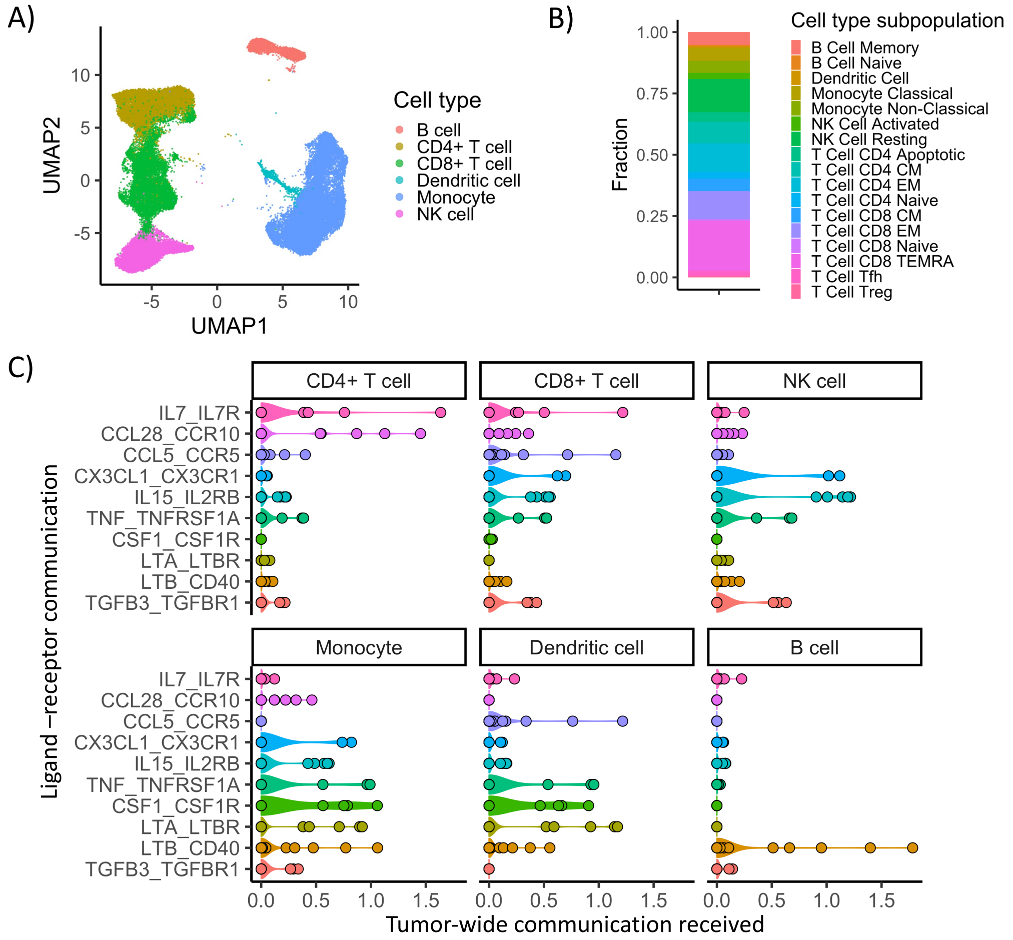

Figure S22) Recovery of known cell type specific communications between immune populations in the peripheral blood using the cell communication analysis. Published scRNAseq data from a study exploring the phenotypic evolution of circulating immune cells of gastrointestinal cancer patients during immunotherapy (GEO accession no. GSE130157) was used to validate that known cell interactions can be recovered. A) The transcriptomic profile of the 70781 cells sampled in the study was used to characterize the phenotype heterogeneity of immune cell types, using UMAP. The phenotypic similarity (little overlap of cell type clusters) within cells annotated as T cells, B cells, monocytes and dendritic cell shows the distinct phenotype of each cell type. B) Using the pre-treatment sample from patient HJD33E we calculated the relative abundance of cell types and the subpopulations of phenotypically distinct cells within each cell type (e.g., naïve, central memory (CM), effector memory (EM) and effector memory cells re-expressing CD45RA (TEMRA) T cells). Distinct cell subtype phenotypes were identified by the authors using differential gene expression analyses. C) The extended expression product method was used to reconstruct the communication between cell type subpopulations using ligand-receptor (LR) gene expression. Specific LR communications (y axis) known to be particularly received by certain cell types were extracted. The extended expression product method inferred the communication strength received by immune cells of the focal cell type (panels=signal receiver) from each cell type population (points). Violin plots show the distribution of communication strengths received from across cell types. As expected, T cells received greater IL7 signals, highly activated effector NK cells received greater signals via CX3CR1, monocytes received greater CSF1 and TNF signals and B cells received greater signals via CD40.

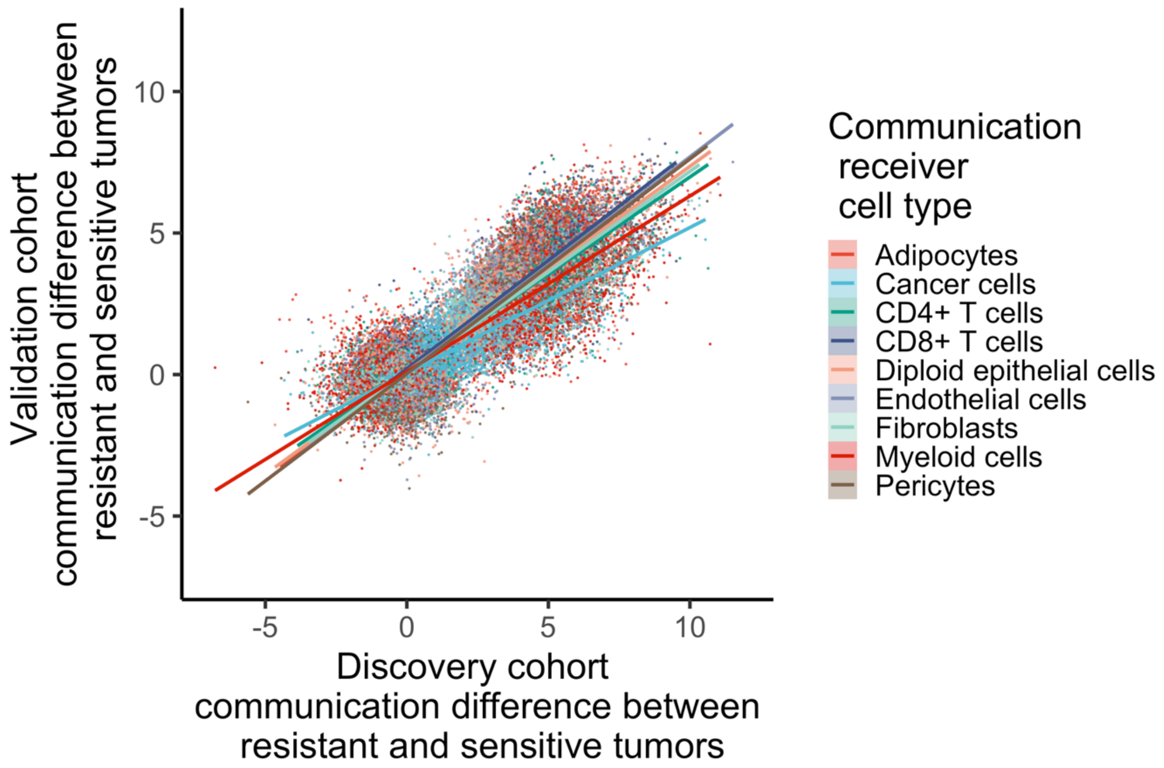

Figure S23) Consistent communication measurements between the independently profiled discovery and validation cohorts) Scatterplot comparison of communication measurements between discovery (x axis) and validation (y axis) cohorts. Each point represents an estimate of the strength of communication from one cell type to a focal receiving cell type (color) via a specific LR communication pathway. Regression lines are plotted showing the consistency of the strength of communication measured in the discovery and validation cohort. A strong and significant correlation of 0.82 was found across cell types (df=106734, t= 679.01, p <0.00001)

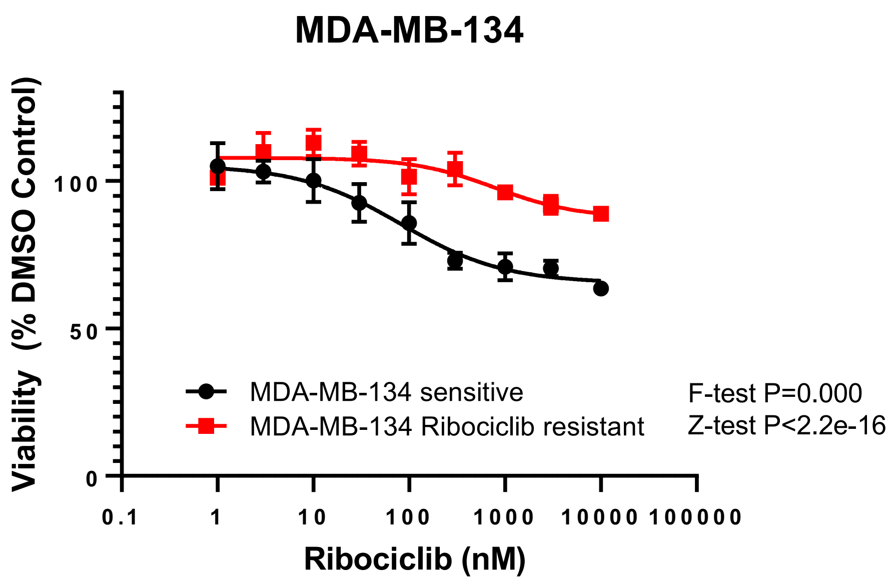

Figure S24) Confirmation of ribociclib cell cycle therapy resistance of experimentally evolved MD-MB-134 cancer cell lines. Dose-response curves of viability of MD-MB-134 ribociclib resistant(red) and sensitive (black) ER+ breast cancer cell lines under ribociclib treatment. Cells were treated with increasing concentration of ribociclib for 4 days and viability was measured using CellTiter-Glo Chemiluminescent kit. Data represents % viable cells compared with DMSO control treatment for each cell line. Points show the mean of four replicates (error bars = mean ± SD). P-values for significance of difference in dose response curves between cell lines were calculated using a 4-parameter log-logistic model and F-test. P-values for differential ED50 values between cell lines were calculated by Z-test. All significance tests were two-tailed.

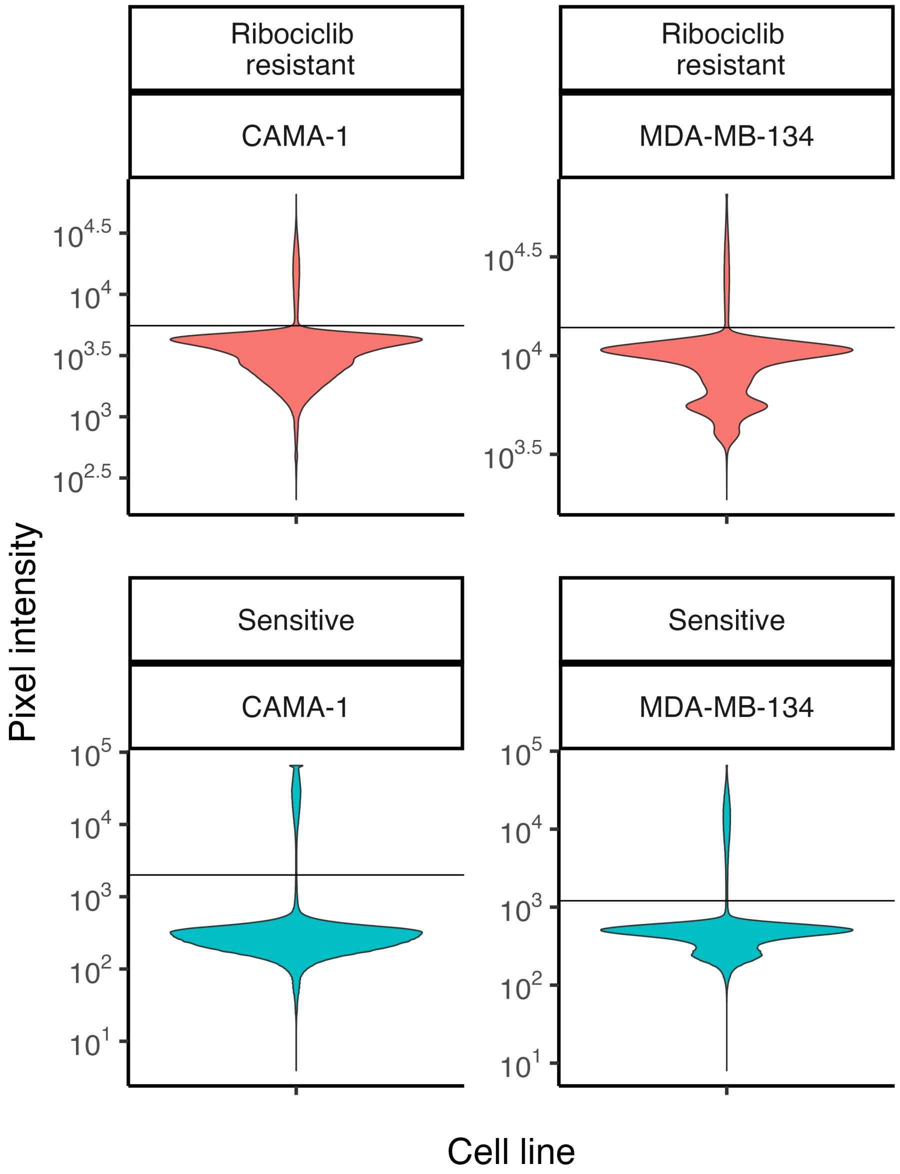

Figure S25) Identified fluorescent cancer cell signal. Violin plot distribution of fluorescence intensities of individual image pixels across experimental timepoints and treatment conditions for each CAMA-1 (Top) and MDA-MB-134 (Bottom) sensitive (right, YFP) and ribociclib resistant (left, CFP FRET V2) cell lines. We randomly sampled 1000 pixels from each experiment image. A fluorescence intensity threshold was then assigned for each cell line (horizontal lines) to distinguish fluorescently labelled viable cancer cells (peaks above the horizontal line) from noise contributed by dead or unlabeled cells and background signal (peaks below the horizontal line). Threshold cutoff values were then used by Gen5 software to quantify viable cancer cell growth for each cell line.
