# Supplementary material for: Cellular interactions within the immune microenvironment underpins resistance to cell cycle inhibition in breast cancers
Source: Nat Commun. 2025 Mar 3;16:2132. doi: 10.1038/s41467-025-56279-x (PMC11876604; doi:10.1038/s41467-025-56279-x)
Supplement: Supplementary file 2 — Description of Additional Supplementary Files [file 41467_2025_56279_MOESM2_ESM.pdf]

Description of Additional Supplementary Files

Supplementary Data 1) Ribociclib treatment communication differences between resistant and sensitive tumors at day 0. Tumor-wide communication analysis was used to measure tumor wide communication sent by one cell type population and received by individual cells of each cell type, via a specific ligand receptor communication pathway (LR). Differences in the strength of communication between a sending and receiving cell type via a specific LR pathway were measured using linear models and ANOVA, with FDR used to account for multiple comparisons. Tests are two-sided.

Supplementary Data 2) Ribociclib treatment communication differences between resistant and sensitive tumors at day 180. Tumor-wide communication analysis was used to measure tumor wide communication sent by one cell type population and received by individual cells of each cell type, via a specific ligand receptor communication pathway (LR). Differences in the strength of communication between a sending and receiving cell type via a specific LR pathway were measured using linear models and ANOVA, with FDR used to account for multiple comparisons. Tests are two-sided.

Supplementary Data 3) Ribociclib treatment communication changes over time seen in resistant tumors. Tumor-wide communication analysis was used to measure tumor wide communication sent by one cell type population and received by individual cells of each cell type, via a specific ligand receptor communication pathway (LR). Differences in the strength of communication between a sending and receiving cell type via a specific LR pathway were measured using linear models and ANOVA, with FDR used to account for multiple comparisons. Tests are two-sided.

Supplementary Data 4) Ribociclib treatment communication changes over time seen in sensitive tumors relative to resistant. Tumor-wide communication analysis was used to measure tumor wide communication sent by one cell type population and received by individual cells of each cell type, via a specific ligand receptor communication pathway (LR). Differences in the strength of communication between a sending and receiving cell type via a specific LR pathway were measured using linear models and ANOVA, with FDR used to account for multiple comparisons. Tests are two-sided.

Supplementary Data 5) Ribociclib treatment communication changes over time seen in sensitive tumors. Tumor-wide communication analysis was used to measure tumor wide communication sent by one cell type population and received by individual cells of each cell type, via a specific ligand receptor communication pathway (LR). Differences in the strength of communication between a sending and receiving cell type via a specific LR pathway were measured using linear models and ANOVA, with FDR used to account for multiple comparisons. Tests are two-sided.

Supplementary Data 6) Validation cohort ribociclib treatment communication differences between resistant and sensitive tumors at day 0. Tumor-wide communication analysis was used to measure tumor wide communication sent by one cell type population and received by individual cells of each cell type, via a specific ligand receptor communication pathway (LR). Differences in the strength of communication between a sending and receiving cell type via a specific LR pathway were measured using linear models and ANOVA, with FDR used to account for multiple comparisons. Tests are two-sided.

Supplementary Data 7) Validation cohort ribociclib treatment communication differences between resistant and sensitive tumors at day 180. Tumor-wide communication analysis was used to measure tumor wide communication sent by one cell type population and received by individual cells of each cell type, via a specific ligand receptor communication pathway (LR). Differences in the strength of communication between a sending and receiving cell type via a specific LR pathway were measured using linear models and ANOVA, with FDR used to account for multiple comparisons. Tests are two-sided.

Supplementary Data 8) Validation cohort ribociclib treatment communication changes over time seen in resistant tumors. Tumor-wide communication analysis was used to measure tumor wide communication sent by one cell type population and received by individual cells of each cell type, via a specific ligand receptor communication pathway (LR). Differences in the strength of communication between a sending and receiving cell type via a specific LR pathway were measured using linear models and ANOVA, with FDR used to account for multiple comparisons. Tests are two-sided.

Supplementary Data 9) Validation cohort ribociclib treatment communication changes over time in sensitive tumors relative to resistant. Tumor-wide communication analysis was used to measure tumor wide communication sent by one cell type population and received by individual cells of each cell type, via a specific ligand receptor communication pathway (LR). Differences in the strength of communication between a sending and receiving cell type via a specific LR pathway were measured using linear models and ANOVA, with FDR used to account for multiple comparisons. Tests are two-sided.

Supplementary Data 10) Validation cohort ribociclib treatment communication changes over time seen in sensitive tumors. Tumor-wide communication analysis was used to measure tumor wide communication sent by one cell type population and received by individual cells of each cell type, via a specific ligand receptor communication pathway (LR). Differences in the strength of communication between a sending and receiving cell type via a specific LR pathway were measured using linear models and ANOVA, with FDR used to account for multiple comparisons. Tests are two-sided.

Supplementary Data 11) Letrozole treatment communication differences between resistant and sensitive tumors at day 0. Tumor-wide communication analysis was used to measure tumor wide communication sent by one cell type population and received by

individual cells of each cell type, via a specific ligand receptor communication pathway (LR). Differences in the strength of communication between a sending and receiving cell type via a specific LR pathway were measured using linear models and ANOVA, with FDR used to account for multiple comparisons. Tests are two-sided.

Supplementary Data 12) Letrozole treatment communication differences between resistant and sensitive tumors at day 180. Tumor-wide communication analysis was used to measure tumor wide communication sent by one cell type population and received by individual cells of each cell type, via a specific ligand receptor communication pathway (LR). Differences in the strength of communication between a sending and receiving cell type via a specific LR pathway were measured using linear models and ANOVA, with FDR used to account for multiple comparisons. Tests are two-sided.

Supplementary Data 13) Letrozole treatment communication changes over time seen in resistant tumors. Tumor-wide communication analysis was used to measure tumor wide communication sent by one cell type population and received by individual cells of each cell type, via a specific ligand receptor communication pathway (LR). Differences in the strength of communication between a sending and receiving cell type via a specific LR pathway were measured using linear models and ANOVA, with FDR used to account for multiple comparisons. Tests are two-sided.

Supplementary Data 14) Letrozole treatment communication changes over time seen in sensitive tumors relative to resistant. Tumor-wide communication analysis was used to measure tumor wide communication sent by one cell type population and received by individual cells of each cell type, via a specific ligand receptor communication pathway (LR). Differences in the strength of communication between a sending and receiving cell type via a specific LR pathway were measured using linear models and ANOVA, with FDR used to account for multiple comparisons. Tests are two-sided.

Supplementary Data 15) Letrozole treatment communication changes over time seen in sensitive tumors. Tumor-wide communication analysis was used to measure tumor wide communication sent by one cell type population and received by individual cells of each cell type, via a specific ligand receptor communication pathway (LR). Differences in the strength of communication between a sending and receiving cell type via a specific LR pathway were measured using linear models and ANOVA, with FDR used to account for multiple comparisons. Tests are two-sided.

Supplementary Data 16) Validation cohort letrozole treatment communication differences between resistant and sensitive tumors at day 0. Tumor-wide communication analysis was used to measure tumor wide communication sent by one cell type population and received by individual cells of each cell type, via a specific ligand receptor communication pathway (LR). Differences in the strength of communication between a sending and receiving cell type via a specific LR pathway were measured using linear

models and ANOVA, with FDR used to account for multiple comparisons. Tests are two-sided.

Supplementary Data 17) Validation cohort letrozole treatment communication differences between resistant and sensitive tumors at day 180. Tumor-wide communication analysis was used to measure tumor wide communication sent by one cell type population and received by individual cells of each cell type, via a specific ligand receptor communication pathway (LR). Differences in the strength of communication between a sending and receiving cell type via a specific LR pathway were measured using linear models and ANOVA, with FDR used to account for multiple comparisons. Tests are two-sided.

Supplementary Data 18) Validation cohort letrozole treatment communication changes over time seen in resistant tumors. Tumor-wide communication analysis was used to measure tumor wide communication sent by one cell type population and received by individual cells of each cell type, via a specific ligand receptor communication pathway (LR). Differences in the strength of communication between a sending and receiving cell type via a specific LR pathway were measured using linear models and ANOVA, with FDR used to account for multiple comparisons. Tests are two-sided.

Supplementary Data 19) Validation cohort letrozole treatment communication changes over time seen in sensitive tumors relative to resistant. Tumor-wide communication analysis was used to measure tumor wide communication sent by one cell type population and received by individual cells of each cell type, via a specific ligand receptor communication pathway (LR). Differences in the strength of communication between a sending and receiving cell type via a specific LR pathway were measured using linear models and ANOVA, with FDR used to account for multiple comparisons. Tests are two-sided.

Supplementary Data 20) Validation cohort letrozole treatment communication changes over time seen in sensitive tumors. Tumor-wide communication analysis was used to measure tumor wide communication sent by one cell type population and received by individual cells of each cell type, via a specific ligand receptor communication pathway (LR). Differences in the strength of communication between a sending and receiving cell type via a specific LR pathway were measured using linear models and ANOVA, with FDR used to account for multiple comparisons. Tests are two-sided.

Supplementary Data 21) Under ribociclib treatment, cancer to myeloid M2 stimulating communications were reduced in sensitive tumors prior to treatment (Day 0). Supervised analysis of the difference in the strength of ligand-receptor (LR) communication from cancer to myeloid cells between resistant and sensitive tumors. The LR communication pathways compared bind M2 differentiation signals which were identified in the communication pathway analysis. Differences in the strength of communication from the

heterogeneous population of cancer cells to myeloid cells via a specific LR pathway were measured using linear models and ANOVA. Tests are two-sided.

Supplementary Data 22) Validation cohort assessment of cancer to myeloid M2 stimulating communication differences between ribociclib resistant and sensitive tumors prior to treatment (Day 0). Supervised analysis of the difference in the strength of ligand-receptor (LR) communication from cancer to myeloid cells between resistant and sensitive tumors. The LR communication pathways compared bind M2 differentiation signals which were identified in the communication pathway analysis. Differences in the strength of communication from the heterogeneous population of cancer cells to myeloid cells via a specific LR pathway were measured using linear models and ANOVA. Tests are two-sided.

Supplementary Data 23) Under letrozole treatment, cancer to myeloid M2 stimulating communications were not reduced in sensitive tumors prior to treatment (Day 0). Supervised analysis of the difference in the strength of ligand-receptor (LR) communication from cancer to myeloid cells between resistant and sensitive tumors. The LR communication pathways compared bind M2 differentiation signals which were identified in the communication pathway analysis. Differences in the strength of communication from the heterogeneous population of cancer cells to myeloid cells via a specific LR pathway were measured using linear models and ANOVA. Tests are two-sided.

Supplementary Data 24) Validation cohort assessment of cancer to myeloid M2 stimulating communication differences between letrozole resistant and sensitive tumors prior to treatment (Day 0). Supervised analysis of the difference in the strength of ligand-receptor (LR) communication from cancer to myeloid cells between resistant and sensitive tumors. The LR communication pathways compared bind M2 differentiation signals which were identified in the communication pathway analysis. Differences in the strength of communication from the heterogeneous population of cancer cells to myeloid cells via a specific LR pathway were measured using linear models and ANOVA. Tests are two-sided.

Supplementary Data 25) Under ribociclib treatment myeloid to T cell immune stimulating communications were consistently activated in sensitive but not resistant tumors. We analyzed at the single cell level how immune activating communications (defined by gene ontology database) from the myeloid population to individual CD8+ T cells diverged during treatment in resistant and sensitive tumors. Hierarchical regression models described pre-treatment differences in CD8+ T cell activating communication between resistant and sensitive tumors and temporal change during treatment. Significant divergence in immune activating communication with T cells of resistant and sensitive tumors was determined using a two-tailed t-test. The Satterthwaite method was applied to perform degree of freedom, t-statistic and p-value calculations.

Supplementary Data 26) Validation in the second independent cohort that under ribociclib treatment myeloid to T cell immune stimulating communications were consistently activated in sensitive but not resistant tumors. We analyzed at the single cell level how immune activating communications (defined by gene ontology database) from the myeloid population to individual CD8+ T cells diverged during treatment in resistant and sensitive tumors. Hierarchical regression models described pre-treatment differences in CD8+ T cell activating communication between resistant and sensitive tumors and temporal change during treatment. Significant divergence in immune activating communication with T cells of resistant and sensitive tumors was determined using a two-tailed t-test. The Satterthwaite method was applied to perform degree of freedom, t-statistic and p-value calculations.

Supplementary Data 27) Under letrozole treatment myeloid to T cell immune stimulating communications were not consistently activated in sensitive versus resistant tumors. We analyzed at the single cell level how immune activating communications (defined by gene ontology database) from the myeloid population to individual CD8+ T cells diverged during treatment in resistant and sensitive tumors. Hierarchical regression models described pre-treatment differences in CD8+ T cell activating communication between resistant and sensitive tumors and temporal change during treatment. Significant divergence in immune activating communication with T cells of resistant and sensitive tumors was determined using a two-tailed t-test. The Satterthwaite method was applied to perform degree of freedom, t-statistic and p-value calculations.

Supplementary Data 28) Validation in the second independent cohort that under letrozole treatment myeloid to T cell immune stimulating communications were not consistently activated in sensitive versus resistant. We analyzed at the single cell level how immune activating communications (defined by gene ontology database) from the myeloid population to individual CD8+ T cells diverged during treatment in resistant and sensitive tumors. Hierarchical regression models described pre-treatment differences in CD8+ T cell activating communication between resistant and sensitive tumors and temporal change during treatment. Significant divergence in immune activating communication with T cells of resistant and sensitive tumors was determined using a two-tailed t-test. The Satterthwaite method was applied to perform degree of freedom, t-statistic and p-value calculations.

Supplementary Data 29) Condensed table of all statistical comparisons of myeloid to T cell activation and recruitment communications across treatments and cohorts. This provides statistical significance of hierarchical random effects model estimates for all comparisons presented in figure 5

Supplementary Data 30) Knowledge-defined myeloid differentiation marker gene list indicating whether the gene is known to support M1-/M2-like differentiation, the published source of this knowledge, a description of the gene's function and an indicator

column denoting whether (1) or not (0) the gene was measured in >5% of cells and was used as a component of the gene signature used to calculate gene set enrichment.
