# Supplementary material for: Cellular interactions within the immune microenvironment underpins resistance to cell cycle inhibition in breast cancers
Source: Nat Commun. 2025 Mar 3;16:2132. doi: 10.1038/s41467-025-56279-x (PMC11876604; doi:10.1038/s41467-025-56279-x)
Supplement: Supplementary file 4 — Reporting Summary [file 41467_2025_56279_MOESM4_ESM.pdf]

nature portfolio

Corresponding author(s): Dr Jason I Griffiths

Last updated by author(s): 12/12/2024

Reporting Summary

Nature Portfolio wishes to improve the reproducibility of the work that we publish. This form provides structure for consistency and transparency in reporting. For further information on Nature Portfolio policies, see our Editorial Policies and the Editorial Policy Checklist.

Statistics

For all statistical analyses, confirm that the following items are present in the figure legend, table legend, main text, or Methods section.

n/a	Confirmed	
☐	☒	The exact sample size (*n*) for each experimental group/condition, given as a discrete number and unit of measurement
☐	☒	A statement on whether measurements were taken from distinct samples or whether the same sample was measured repeatedly
☐	☒	The statistical test(s) used AND whether they are one- or two-sided<br>*Only common tests should be described solely by name; describe more complex techniques in the Methods section.*
☐	☒	A description of all covariates tested
☐	☒	A description of any assumptions or corrections, such as tests of normality and adjustment for multiple comparisons
☐	☒	A full description of the statistical parameters including central tendency (e.g. means) or other basic estimates (e.g. regression coefficient) AND variation (e.g. standard deviation) or associated estimates of uncertainty (e.g. confidence intervals)
☐	☒	For null hypothesis testing, the test statistic (e.g. *F*, *t*, *r*) with confidence intervals, effect sizes, degrees of freedom and *P* value noted<br>*Give P values as exact values whenever suitable.*
☒	☐	For Bayesian analysis, information on the choice of priors and Markov chain Monte Carlo settings
☐	☒	For hierarchical and complex designs, identification of the appropriate level for tests and full reporting of outcomes
☐	☒	Estimates of effect sizes (e.g. Cohen's *d*, Pearson's *r*), indicating how they were calculated

Our web collection on statistics for biologists contains articles on many of the points above.

Software and code

Policy information about availability of computer code

Data collection	Data were generated at the City of Hope Cancer Center.
Data analysis	Custom code used in analyses are available on GitHub at https://github.com/U54Bioinformatics/FELINE_project/FELINE_immune_communication.<br><br>Single nuclei RNA sequencing and processing<br>Tumor single cell nuclei were isolated from OCT embedded core tumor biopsies using a modified lysis buffer containing 0.2% Igepal CA-630 as previously described (90). Single cell RNA-Sequencing (scRNAseq) was performed on single nuclei suspensions using 10X Genomics Chromium platform as previously described (10). Sequence reads were processed with BETSY and CellRanger v3.0.2, which aligned reads to reference genome (GRChg38) using STAR v2.6.0 (91). For each sample, a gene-barcode count matrix was generated containing counts of unique molecular identifiers (UMIs) for each gene in each cell.<br><br>We reanalyzed the validation cohort cells to recover intermediate-quality non-cancer cells that were excluded based on the filters used originally in the discovery cohort analysis. Cells were clustered based on the percentage of mitochondrial genes using k-means clustering (k =4). We filtered out high mitochondrial content clusters (centroids = 55 and 87% mitochondrial genes) and retained low percent mitochondrial genes (centers = 0.2 and 20%). We further filtered out cells classified as epithelial by SingleR analysis(33) and with less than 100 genes expressed.<br><br>Cell type classification and verification<br>We obtained transcriptional profiles of 424,581 single cells, using stringent quality controls to ensure high-coverage, low mitochondrial content, and doublet removal (as described in (10)). On average, we recovered 2.75 (out of 3) time point samples per patient. Broad cell types

were annotated using singleR(33), cancer cells were identified by their frequent and pronounced copy numbers amplification using inferCNV(34). Cell type annotations were verified by cell type specific marker gene expression and UMAP/TSNE analyses (10, 35). Granular immune subtype annotations were obtained using our recently published ImmClassifier machine learning method, which has been validated by flow cytometry comparisons (36).

Machine learning classifier for cell type annotations
Cell subtype annotations for the discovery and validation cohorts were consistently annotated by training a random forest machine learning classifier to identify cell types using the well-curated discovery cohort data. We then applied the classifier to predict cell type annotations in the validation cohort. First, we identified the marker genes associated with each cell type in the discovery cohort cells, using a negative binomial test to find genes differentially expressed in each cell type relative to all others. Genes expressed in > 25% of cells in at least one group and showing a log fold change in expression > 0.25 between the groups were selected as candidate markers. We additionally included cell cycle score (G2M and S scores calculated by Seurat's CellCycleScoring function) as latent variables. The top 100 marker genes of each cell type were selected as candidate features in the machine learning analysis. The classifier was constructed using SingleCellNet, a top pair random forest approach to predict each cell type (92). First, we split the high-quality C1 cohort into a training and validation subset. We then trained the classifier on the training subset with the parameters nTopGenes = 25, nRand = 100, nTrees = 1000 and nTopGenePairs = 50. Performance assessment in the held-out validation subset showed good performance with area under the receiver operator curve > 0.9 (Figure S2). To confirm the consistency of the discovery and validation cohorts, all cells were projected into a common UMAP space, using the first 10 principal components of the scaled expression levels of 100 marker genes associated with each cell type (Figure S2). We verified that the UMAP clusters, indicating a major biological cell type, were assigned consistent cell type annotations using the across cohorts using the manual curation and machine learning classification approaches. Most cell type were uniquely assigned to a single cluster and this accuracy was further improved by retaining cells with a cell type prediction probability > 0.75.

Archetypal tumor compositions
The composition of each tumor sample was summarized by calculating the proportion of each cell subtype to correct for sampling variation. The compositional similarity of each tumor samples was measured using the pairwise logit-Euclidean distance. This quantified the fraction of each tumor composition that would need to be altered to generate the compositional profile of each other sample. Compositionally similar tumors and collections of cell types with correlated abundances were grouped using hierarchical clustering (method= 'ward.D2').

To identify archetypal ER+ breast cancer tumor compositions, we projected all tumor samples into a composition landscape, using UMAP (version 0.2.3.1) to account for the non-linearity and non-normality of compositional data (35). Highly compositionally similar tumors located close together in this ordination space and distant from tumors with divergent compositions. We then applied a Gaussian Mixture model (GMM) and Bayesian information criterion to probabilistically identify distinctly similar clusters of tumor samples (93). This identified the appropriate number of archetypal tumor compositions supported by the data and classified each tumor sample into an archetype. Major compositional differences between archetypal compositions were identified using Dirichlet regression (R package 'DirichletReg' v0.7-1) and the rank correlation of UMAP TME composition axes with cell type frequencies.

Subclonal cancer composition and evolution from scRNA copy number alteration
Cancer subclonal populations of each tumor sample were identified through 'infercnv' analysis (R package 'infercnv' v1.0.2; cutoff=0, min_cells_per_gene=100 or 500, cluster_by_groups=T, HMM=T, analysis_mode= "subclusters"). Genomic regions of copy number alteration in each cell were detected relative a subset of 500 reference immune or stromal cells, using the count matrix. Then, cancer populations with distinct copy number profiles were defined as cancer subclones of a patient tumor using hierarchical clustering (R package 'fastcluster' v1.1.25; method='ward.D2')(94). Clusters with distinct copy number profiles were defined as subclones for each patient. Single-cell grouping was performed based on hierarchical cluster analysis.

Cell phenotypes from Gene Set enrichment analysis
The gene expression count matrix of each cell type was filtered to keep genes expressed in at least 10 cells, zinbwave normalized with total number of counts, gene length and GC-content as covariates (R package 'zinbwave' v 1.8.0; K=2, X="~log (total number of counts)", V="~ GC-content + log (gene length)", epsilon=1000, normalizedValues=TRUE) (95). Single sample Gene Set Enrichment Analysis (ssGSEA) scores of 50 hallmark signatures (MSigDB, hallmark) and 4725 curated pathway signatures (MsigDB, c2) were calculated for each cell using the normalized count matrix in GSVA (R package 'GSVA' v1.30.0; kcdf="Gaussian", method='ssgsea') (96, 97).

Communication from across diverse cell type populations through: Tumor-wide integration of signaling to each receiver cell.
Networks of communication from across diverse cell type populations and received by individual cancer and non-cancer cells of a tumor were uncovered by applying an extended expression product method to ligand-receptor scRNAseq data. This measures population level communication using single-cell gene expression (count per million). We first extended individual level cell-cell interaction (CCI) approaches (reviewed in (22)) to measure communications received from entire cell type populations or from across the entire tumor population (tumor-wide communication), accounting for tumor composition and within cell type phenotypic heterogeneity. The tumor-wide communication metric was derived by formulating a differential equation model of tumor ecosystem signaling. This describes the change in concentration of a signaling ligand molecule (S) in the TME as following:

$$dS/dt=\sum_i (\sigma x)_i P_i - \sum_j q_j (y_j \gamma P)_j S - \mu S.$$

Signals are produced by cells in the TME at a rate proportional to their expression of the signaling ligands. Within cancer and non-cancer cell types, subpopulations vary phenotypically and differ in ligand gene expression, with subpopulation i having ligand expression x_i. Ligands produced by a cell are released into TME at rate σ. The total signal production by each cell subpopulation is proportional their abundance in the TME (P_i) and the total signal production across the TME is given by the sum of production across all cell subpopulations. Signaling ligands are removed from the TME through decay or diffusion at rate (μ) or when bound to a receptor on a receiving cell (receptor binding rate=γ) and taken up (ligand internalization rate= q_j). Phenotypically different subpopulations within cancer and non-cancer cell types have differing receptor concentrations, with receptor density of cell type j depending on its receptor gene expression (y_j). The total ligand uptake by each cell subpopulation is proportional to their abundance in the TME (P_j) and the fraction of molecules are taken up and removed from the TME once receptor bound. The total signal uptake across is given by the sum of uptake across all cell subpopulations.

The steady state analysis of the TME signal concentration is given by:

$$S^*=(\sum_i (\sigma x)_i P_i)/(\sum_j q_j (y_j \gamma P)_j + \mu).$$

Assuming ligand release after receptor binding (q_j is small), the strength of signal transmitted from all cell subpopulation in the TME (tumor-wide communication) to a focal receiver cell in subpopulation j is given by:

〖C_j (x,y_j)= y_j γS〗 ^*= (γσ/(μ)) 〖 y〗 _j ∑_i▒ 〖x_i P_i . 〗

Given a sampled tumor composition ((P_i) ̂=cell proportion of subpopulation i), the tumor-wide communication transmitted via ligand-receptor pathway k to a receiver cell (of type j) can be measured given a vector of ligand gene expression for each cell types present (x_k), and the receptor expression of the receiving cell (y_jk) as: C_jk (x_k,y_jk) ∝ (y_jk ∑_i▒ 〖x_ik (P_i) ̂ 〗) (Figure 1C).

This generalizes the CCI approaches using the ligand-receptor product and expression correlation method (e.g., CCCExplorer, ICELLNET, NATMI, NicheNet and scTensor; reviewed in (22)) to the broader tumor ecosystem perspective. Rather than measuring one-one interactions between individual cells, C_jk (x_k,y_jk) measures the many-one communication strength a focal cell receives from the diversity of cells that are releasing communications into the TME. This is again distinct from the many-many mapping of communication implemented to quantify the probability of cell-cell communication between two cell types (e.g., in CellChat)(24). The extended expression product method therefore allows an assessment of how phenotypically diverse populations of cells contribute communications to the signaling reservoir in the TME to stimulate the receiver cell, accounting for the abundance and ligand production of each signaling phenotype (Figure 1C).

We validated that by restricting tumor-wide communications to individual level communications between one sending cell of one cell type and another receiving cell, measurements are consistent with individual level cell-cell interactions obtained using the ligand-receptor correlation/expression product method (3) (as used in CCCExplorer, ICELLNET, NATMI, NicheNet and scTensor; reviewed in (22)). The model also shows how tumor-wide communications generalize the established CCI approach to the broader tumor ecosystem perspective. Crucially, instead of just revealing how an individual cell of one cell type communicates with a cell of another type (individual one-one cell crosstalk), the extended expression product method allows an assessment of how a phenotypically diverse population of cells within each major cell type (e.g., macrophages in distinct states) contribute communications to the receiver, accounting for the abundance and ligand production of each signaling phenotype (population many-one cell crosstalk). This is distinct from methods such as CellChat which use cell counts to weight the probability that an individual of the two cell types interact (i.e., frequency of individual one-one cell crosstalk).

Measuring tumor-wide communications received from diverse cell phenotypes
We applied the extended expression product method to measure tumor-wide signaling from diverse non-cancer cell sub-populations and heterogeneous cancer lineages to receiving cells. We first resolved diverse subpopulations of each cancer and non-cancer cell type (e.g., macrophages in different differentiation states). For each broad cell type we generated a cell-type specific UMAP based on ssGSEA profiles, with the intrinsic UMAP dimensionality determined using the packing number estimator(98). We then break down each cell type into subtypes of at least 30 cells with coherent phenotypes and of equal interval width along each phenotype axis. This allowed cell types with relatively continuous phenotypic variation, such as macrophages, to be subdivided into an ordered set of cell states along multiple axes of phenotypic heterogeneity and maintains phenotype covariance structure. We then calculated the relative abundance of each subpopulation of each major cell type within a tumor sample ((P_i) ̂).

We next used a curated LR communication database (99) to define a set of 1444 LR communication pathways (C_jk (x_k,y_jk)) based on known protein-protein interactions. We extracted single-cell expression of a ligand and used mean CPM of a cell subpopulation as a metric of signal production (x_k) and the mean CPM receptor expression to quantify signal receipt by a focal cell (y_jk). We calculated activity of each LR communication pathway (k=1:1444) between each pair of sending (i) and receiving (j) subpopulations (i→j) within a tumor: C_(i→j,k) (x_ik,y_jk)=(y_jk x_ik (P_i) ̂).

Strength of communication between cell types: contribution and receipt of signals
To obtain communications via LR pathway k between broad cell types, we totaled signals from sending cell type populations (1:n ligand producing subpopulations of a cell type) and averaged signals to receiving cells (across 1:m signal receiving subpopulations of a cell type). We use a weighted average so that the signal to each receiving cell type population is weighted by abundance (Figure 1C):
C_(i_(1:n)→j ̅,k) (x_(i_(1:n) k),y_(〖j 〗 _(1:m) k))= (∑_(z=1)^m▒(C_(i_(1:n)→ 〖j 〗 _z k) (x_(i_(1:n)),y_(〖j 〗 _z k)) P ̂_(〖j 〗 _z)))/(∑_(z=1)^m▒((P_(〖j 〗 _z)) ̂)).

We refer to this as the strength of communication from a cell type population to a typical cell of another type. For example, the contribution of n heterogeneous cancer cell populations (〖Cancer〗 _(1:n)) to the communication with a myeloid cell of phenotype z via ligand x and receptor y is given by:
C_(〖Cancer〗 _(1:n)→ 〖Myeloid 〗 _z,k) (x_(〖Cancer〗 _(1:n) k),y_(〖Myeloid 〗 _z k))= 〖 y〗 _(〖Myeloid 〗 _z k) ∑_(i=1)^n▒ 〖x_(〖Cancer〗 _i k) P ̂_(〖Cancer〗 _i) 〗

The strength of communication received by a typical myeloid cell in a sample ((Myeloid) ̅) from the diversity of cancer cells is given by:
C_(〖Cancer〗 _(1:n)→(Myeloid) ̅,k) (x_(〖Cancer〗 _(1:n) k),y_(〖Myeloid 〗 _(1:m) k))=
(∑_(z=1)^m▒(C_(〖Cancer〗 _(1:n)→ 〖Myeloid 〗 _z,k) (x_(〖Cancer〗 _(1:n) k),y_(〖Myeloid 〗 _z k)) P ̂_(〖Myeloid 〗 _z)))/(∑_(z=1)^m▒((P_(〖Myeloid 〗 _z)) ̂))

This was repeated for each LR communication pathway between cell types and across tumor samples. Each communication pathway has a distinct potency to modulate cellular phenotype and behavior and so communication pathway scores were standardized (mean=0, sd=1) across patients, preventing highly expressed ligand-receptor pairs dominating communications. The average strength of communication from one cell type to another across LR pathways (C ̅_(i_(1:n)→j ̅)) was measured by the median standardized communication from one cell to another.

Validation of communication measurements
We validated the method in peripheral blood immune cells, in which communications between cell types are well known and distinctly different from those expected in tumor biopsy samples (100). Our approach successfully recovered the expected communication network, with myeloid cells having a central role in communicating via cytokine pathways with many cell types (Figure S20). We also validated that we could recover canonical cell type specific communications including receipt of: macrophage colony stimulating factor primarily in myeloid cells, vascular endothelial growth factor (VEGF) in endothelial cell, fibroblast growth factor (FGF) in fibroblasts, epidermal growth factor (EGF) in epithelial cells and C-C chemokine receptor type 5 (CCR5) in T cells (Figure S5). Finally, we compared the measured differences in

communication between the resistant and sensitive tumors of the discovery and validation cohort. This verified the high degree of consistency in the signals each cell type received via each LR communication pathway tumor response groups across the two cohorts (R^2=0.81) (Figure S21).

Cell type communication differences between resistant (growing) and sensitive (shrinking) tumors: Bootstrapping randomization comparison
Contrasting communication across many biopsies, rather than within individual samples, provided comparative insights into the evolution of communication during treatment and the cell type communications that distinguish resistant and sensitive tumors. We contrasted the networks of communication between cell types in resistant and sensitive tumors and examined how communications changed throughout treatment with ribociclib or letrozole. We determined the difference in the average strength of communication from one cell type to another ($\bar{C}_{(i_{(1:n)}\rightarrow \bar{j})}$) between resistant and sensitive tumors. To identify which cell type communications significantly differed between tumors resistant and sensitive to each treatment, we perform a bootstrapping randomization analysis. We repeatedly shuffled the observed cell type L-R communications across resistant and sensitive tumors to remove any response related structure of the communication network. For 1000 randomized communication networks, the difference in average communication was recalculated. The distribution of communication differences produced by chance in the randomized networks (null model: average communication does not differ between resistant and sensitive tumors) was then compared to the observed difference in the average communication between cell types.

Using the mean and standard deviation of the communication differences in the randomized networks, z statistics were calculated to indicate how much each cell type's communication differed between resistant and sensitive tumors. Randomization p-values were calculated by the rank of the observed average communication difference within the distribution of randomized differences between resistant and sensitive communication networks. A Holm's conservative correction for statistical significance was applied to correct for multiple comparisons.

Divergent communication networks between cell types in resistant (growing) and sensitive (shrinking) tumors
We obtained the expected cell type communication of one cell type to another in resistant and sensitive tumors at each time point of each treatment. This summarized the average strength of communication ($\bar{C}_{(i_{(1:n)}\rightarrow \bar{j})}$) across each individual tumor within each response category and treatment time point. Pre-treatment cell type communication networks were then described by directed weighted network graphs, constructed for resistant and sensitive tumors. Cell types were represented by network nodes and the proportional changes in communication were described by the weight of the vertex from one cell type to another (indicated by arrow width). We also calculated the proportional change in expected cell type communication post treatment in resistant and sensitive tumors, relative to the baseline overall average.

Communication pathway analysis: identifying response related communications
For each LR communication pathway, we contrasted the strength of communication between cell types in tumors growing (resistant) or shrinking (sensitive) during each treatment. We used log-linear regression to describe trends in cell type communication within resistant and sensitive tumors ($\log(1+C_{(i_{(1:n)}\rightarrow \bar{j},k)}(x_{(i_{(1:n)}k)},y_{(j_{(1:m)}k)})))$). General communication trends during treatment and changes specific to growing treatment-resistant tumors were detected using likelihood ratio tests. Significant differences in the strength of cell type communication between resistant and sensitive tumors either before or after treatment were identified by using ANOVA on the endpoint data (Day 0 and 180 separately). We accounted for multiple comparisons using false discovery rate (FDR) p-value correction. To identify broadly divergent communications between resistant and sensitive tumors before treatment, we enumerated the detected communications sent and received by each cell type.

Myeloid phenotype reconstruction
The diversity of myeloid phenotypes was examined through UMAP analysis of single cell transcriptional profiles (log(1+CPM)). Genes with greater than 5% coverage in cells were used. Dendritic cell and macrophage cell subtype annotations obtained from ImmClassifier (36) were overlaid onto the UMAP, confirming consistent identification of distinct cell population between approaches. The M1-like:M2-like phenotype gradient across the UMAP (dimension 2) was identified using the rank correlation of UMAP axes each genes expression. To test for pre-treatment differences in macrophage polarization between growing treatment-resistant and shrinking treatment-sensitive tumors, we fitted a hierarchical linear model describing the M1-like:M2-like phenotype score differed in myeloid cells from resistant and sensitive tumors, accounting for patient specific heterogeneity in myeloid phenotype and the shared TME of cells within a sample. Finally, M1-like macrophages were defined as having less average M1-like:M2-like phenotype scores and other macrophages classified into the M2-like phenotype.

Verifying that myeloid polarization predicts resistance
We next confirmed that ribociclib-resistant tumors can be identified early in treatment (Day 0-14) by the increased M2-like differentiation of their myeloid cells. We contrasted the phenotypes of myeloid cells in resistant and sensitive tumors in the independently profiled verification cohort.
We characterized the phenotypic heterogeneity of myeloid cells in the verification cohort. We used the fitted UMAP model, trained with the discover cohort CPM data, to project myeloid cells of the validation cohort into a consistent myeloid phenotype space. This provided equivalent M1-like:M2-like phenotype scores for each myeloid cell of the validation cohort.

We then assessed whether myeloid cells of growing ribociclib-resistant tumors showed greater M2-like differentiation in the validation cohort. We applied three complementary analyses. First, we applied a hierarchical linear model to test for differences in single cell M1-like:M2-like phenotype scores between resistant and sensitive tumors throughout treatment, accounting for patient specific heterogeneity in myeloid phenotype. Second, we summarized the mean M1-like:M2-like phenotype score of myeloid cells in each tumor. We then contrasted the mean myeloid differentiation early in treatment (Day 0-14) between resistant and sensitive tumors using ANOVA. Finally, we compared the relative abundance of M1-like and M2-like macrophages in resistant and sensitive tumors early in treatment, using logistic regression to describe how the proportion of M1-like cells per tumor biopsy varied by treatment and resistance outcome.

Measuring targeted cancer cell signaling to M1-like and M2-like macrophages
We identified cancer cell communications that predominantly target either M1-like or M2-like macrophages. First, we measured cell-cell interactions between phenotypically diverse cancer and macrophage subpopulations in each tumor sample, using the ligand-receptor product approach (22). For each tumor sample, we calculated the average cell-cell interaction of cancer cells with each macrophage via each communication pathway. We contrasted the log cancer-macrophage cell-cell interaction received by M1-like and M2-like macrophages, using a hierarchical linear model to detect differential communication with myeloid cell types and to account for baseline tumor specific differences cancer-macrophage communication. Significant differences in cancer communication with M1-like and M2-like cells were identified using likelihood ratio tests contrasting: i) the full model with communication to M1-like and M2-like cells differing and ii) the nested null model with no difference in communication. The twenty most significantly activated communications with M1-like and M2-like macrophage were assessed.

Contrasting the heterogeneity of cancer to macrophage communications across resistant and sensitive tumors
We combined the list of communication pathways through which cancer cells: a) communicate more strongly with macrophages in resistant than sensitive tumors (see Communication pathway analysis) and b) have stronger cell-cell interactions with M2-like versus M1-like macrophages (as described above). From this list, we identified the ligands the cancer cells used to modulate macrophage phenotype and tumor response. Communication pathways binding these ligands were defined as M2-like differentiation communications and selected for supervised analysis. We contrasted the strength of communication from cancer to myeloid cells via each M2-like differentiation communication pathway in resistant and sensitive tumors samples taken early in each treatment (Day 0 and 14). Heatmaps were used to visualize the heterogeneity of communication pathway activity across tumors.

Cancer and non-cancer cell type contributions to myeloid polarizing communications
We next determined which cell types most strongly contributed to myeloid differentiation communications. We extracted the strengths of each M2-like differentiation communication sent from each cell type to myeloid cells. For each tumor sample, the median standardized M2-like differentiation communication from each cell type was calculated. We then contrasted the average M2-like differentiation communication sent by each cell type in resistant and sensitive tumors and under each treatment.

Identifying differential communications of M1-like and M2-like myeloid cells with CD8+ T cells
We next identified myeloid communications with T cells primarily produced by either M1-like or M2-like myeloid cells. We first measured cell-cell interactions between phenotypically diverse macrophage and T cell subpopulations in each tumor sample, using the ligand-receptor product approach (22). For each tumor sample, we calculated the average cell-cell interaction of M1-like and M2-like myeloid cells with T cells via each communication pathway.

We then identified communication pathways by which T cells received significantly different cell-cell interactions from M1-like and M2-like myeloid cells. The log macrophage-T cell interactions from M1-like and M2-like macrophages were contrasted, using hierarchical linear models. A patient specific random component accounted for the heterogeneity in immune communication between TME's and a random component associated with T cell phenotype accounted for the diversity of T cell activation phenotypes within and between tumors. Significant differences in T cell communication from M1-like and M2-like cells were identified using likelihood ratio tests contrasting: i) the full model with communication from M1-like and M2-like cells differing and ii) the nested null model with no difference in communication.

Contrasting M1-like communication with T cells in resistant and sensitive tumors
Next, we isolated the M1-like macrophages and examined their communication with T cells in resistant and sensitive tumors. For each communication pathway, we contrasted M1-like macrophage to T cell interactions (log transformed) in resistant and sensitive tumor using hierarchical linear models. Again, a patient specific random component accounted for the heterogeneity in immune communication between TME's and a random component associated with T cell phenotype accounted for the diversity of T cell activation phenotypes within and between tumors. A likelihood ratio test was used to detect communication pathways significantly differing between resistant and sensitive tumors.

Diverging inflammatory communication from myeloid cells to CD8+ T cells in resistant and sensitive tumors
We next determined how the M2-like polarization of the myeloid population in ribociclib-resistant tumors impacted the communication of immune cytokine signals to T cells. First immune activating inflammatory cytokine communications were identified, using the receptors gene-ontology database signatures (66). For each CD8+ T cells we totaled the signal received from all myeloid subpopulations within that tumor sample via each inflammatory cytokine communication pathway. To obtain the overall immune activating communication received by each CD8+ T cell from the myeloid population, we averaged across communications pathway scores after scaling and log transformation.

We then analyzed at the single cell level how each of the immune activating communications from myeloid to CD8+ T cells diverged during treatment in resistant and sensitive tumors. Using a hierarchical regression model, we described pre-treatment differences in CD8+ T cell activating communication between resistant and sensitive tumors and temporal change during treatment (as previously described in (10)). Significant divergence in immune activating communication with T cells of resistant and sensitive tumors was determined using a two-tailed t-test. The Satterthwaite method was applied to perform degree of freedom, t-statistic and p-value calculations, using the 'lmerTest' R package (v3.1-3) (101).

Linking myeloid inflammatory communications to CD8+ T cell activation
We characterized how the differentiation and activation to an effector CD8+T cell phenotype was related to the strength of immune cytokine communication they received from myeloid cells. For each CD8+ T cell, differentiation was measured using a CD8+ T cell specific ssGSEA pathway contrasting gene expression of naive and cancer killing effector cells (GSE 22886 Naive CD8 T cell vs NK cell up). The single cell activation state was then linked to the immune activating communication received from across the myeloid population (measured above). Each CD8+ T cells differentiation state was then linked to the inflammatory cytokine communication it received from across the myeloid population. Linear regression was used to measure the increase in T cell activation with increasing inflammatory communication. The strength of inflammatory cytokine communication received was discretized into deciles of signal strength and the distribution of phenotypic state assessed in cells receiving each level of stimulus.

We then analyzed at the single cell level how the CD8+ T cell activation diverged during treatment in resistant and sensitive tumors. Using a hierarchical regression model, we described pre-treatment differences in CD8+ T cell activation between resistant and sensitive tumors and temporal change during treatment (detailed in (10)). Significant divergence of CD8+ T cell activation in resistant and sensitive tumors was determined using a two-tailed t-test. The Satterthwaite method was applied to perform degree of freedom, t-statistic and p-value calculations, using the (101).

Contrasting T cells relative abundance during treatment in resistant and sensitive tumors
Differences in T cell abundance between resistant and sensitive tumors were analyzed at each treatment time point and separately for tumors receiving each treatment, using logistic regression. We identified significant differences in the proportion of T cells between tumor response groups using a two-tailed Wald-test to generate z statistics and p values.

Comparing post treatment immune response across cell types in resistant and sensitive tumors
We compared the difference in immune response observed across all cell types between tumors resistant and sensitive to each treatment. For each single cell observed at the end of treatment, we measured immune stimulation using Hallmark Interferon Gamma response ssGSEA pathways.

We analyzed the difference in Interferon Gamma response between treatment-resistant and sensitive tumors, using a nested hierarchical regression model to account for the patient specific differences in immune response and the between cell type differences in this phenotype. Cell type specific random effects were nested within the patient random component, reflecting the occurrence of each cell type within different patient tumors. Significant divergence in Interferon Gamma response between resistant and sensitive tumors treated with combination ribociclib or letrozole alone were determined using two-tailed t-tests.

We then analyzed at the single cell level how the CD8+ T cell activation diverged during treatment in resistant and sensitive tumors. Using a hierarchical regression model, we described pre-treatment differences in CD8+ T cell activation between resistant and sensitive tumors and temporal change during treatment (described in (10)). Significant divergence of CD8+ T cell activation in resistant and sensitive tumors was determined using a two-tailed t-test, again using Satterthwaite method.

Linking inflammatory communications to cancer interferon gamma response
We next determined the how cancer cell phenotypes responded to increasing inflammatory cytokine communications in the TME. We assessed the cancer cells interferon response phenotype, using their hallmark interferon gamma response ssGSEA scores. This cancer phenotype measured intracellular transduction of cytokine signals to the nucleus, induction of interferon regulatory factors (IRFs), Interferon-stimulated gene activation (e.g. Interferon gamma-induced proteins) and the production of antigen presenting major histocompatibility complex molecules (MHC I) allowing recognition and killing of cancer cells (69, 70).

To determine the major communication pathways stimulating a cancer cell interferon gamma response, we next calculated the strength of the communication each cancer cell received from across the TME via each ligand receptor pathway. For each cancer cell, we coupled the single cell interferon response phenotype to the total communication stimulus each cancer received from across the TME via each receptor. The cancer cell data was subset by subclonal cancer genotype (identified in (10)) and communication scores were square root transformed, scaled and centered (mean=0, sd=1) to improve normality and comparability respectively.

For each cancer subclone of each tumor sample, we identified communications strongly associated with activation of an interferon response, using a lasso penalized likelihood regression model (R package 'glmnet' v4.1-8). The lasso penalty (α=1) encourages detection of the communications most strongly activating interferon response, through a shrinkage of the coefficients of all but dominant communications predictors. This variable selection approach minimizes overfitting when considering the role of many communication pathways and enhances the interpretability and predictive accuracy of the model.

Cross-validation (internal 10-fold) was performed to determine the penalty parameter (λ) that minimized the mean cross-validated error. The contribution of each communication to (coefficients) the explained variance in cancer cell interferon phenotype was then assessed. We identified the communication receptors of cancer cells detected to contribute to the interferon phenotype in more than 10% of tumor subclones.

The association of a cancer interferon response with the most communication via the most frequently detected receptor (IL-15RA) was examined using a generalized additive model with a unique smoothing term for resistant and sensitive tumors given each treatment. The IL-15 communication received by cancer cells was also discretized into deciles of signal strength and the distribution of cancer interferon response phenotypes compared to the signal received.

For manuscripts utilizing custom algorithms or software that are central to the research but not yet described in published literature, software must be made available to editors and reviewers. We strongly encourage code deposition in a community repository (e.g. GitHub). See the Nature Portfolio guidelines for submitting code & software for further information.

Data

Policy information about availability of data

All manuscripts must include a data availability statement. This statement should provide the following information, where applicable:

- Accession codes, unique identifiers, or web links for publicly available datasets
- A description of any restrictions on data availability
- For clinical datasets or third party data, please ensure that the statement adheres to our policy

Raw single cell RNA-seq data are available through GEO under accession code GSE211434 at https://www.ncbi.nlm.nih.gov/geo/query/acc.cgi?acc= GSE211434. The following secure token has been created to allow review of record GSE211434 while it remains in private status: axujqkyupluxbmp. Source data provided with this paper include PBMC data and in vitro T cell coculture cancer growth data collected under ribociclib and IL-15 treatment.

Research involving human participants, their data, or biological material

Policy information about studies with human participants or human data. See also policy information about sex, gender (identity/presentation), and sexual orientation and race, ethnicity and racism.

Reporting on sex and gender	We studied the tumor-wide communication among cells in tumors of post-menopausal women with node positive or >2 cm ER+ and/or PR+, HER2 negative breast cancer enrolled on the FELINE clinical trial (10, 31, 32)(clinicaltrials.gov # NCT02712723). Consequently, no sex- and gender-based analyses have been performed or required.
Reporting on race, ethnicity, or other socially relevant groupings	Molecular characteristics of patient tumors and the microenvironment were studied rather than catagorising individuals by race or ethnicity.<br><br>As the study focused on ER+ breast cancer in most-menopausal women (the most common population to experience the

disease), sex and gender was not further considered in the study design. The sex and/or gender of participants was determined based on clinical physiological assessment.

Population characteristics	Estrogen positive breast cancer patients were selected for this study. We studied the tumor-wide communication among cells in tumors of post-menopausal women with node positive or >2 cm ER+ and/or PR+, HER2 negative breast cancer enrolled on the FELINE clinical trial (10, 31, 32)(clinicaltrials.gov # NCT02712723). All patients received neoadjuvent treatment for 180 days. Patients were randomized equally across three treatment arms (40:40:40). Arm A received letrozole plus placebo, Arm B letrozole plus ribociclib 600 mg daily for 21 out of 28 days of each cycle and Arm C received letrozole plus ribociclib 400 mg continuously. Protocol therapy was continued until the day before surgery. Tumor response to treatment was assessed using multiple imaging modalities.
Recruitment	Patients were recruited for the clinical trial if they met the inclusion criteria.
Ethics oversight	University of Kansas (IRB number: CLEE011XUS10T)

Note that full information on the approval of the study protocol must also be provided in the manuscript.

Field-specific reporting

Please select the one below that is the best fit for your research. If you are not sure, read the appropriate sections before making your selection.

☒ Life sciences ☐ Behavioural & social sciences ☐ Ecological, evolutionary & environmental sciences

For a reference copy of the document with all sections, see nature.com/documents/nr-reporting-summary-flat.pdf

Life sciences study design

All studies must disclose on these points even when the disclosure is negative.

Sample size	No sample size assessment was performed for the scientific endpoints.
Data exclusions	Discovery and validation cohort sequencing<br>The 120 patients were divided into two equally sized cohorts: a hypothesis generating discovery cohort and a validation cohort. Two-thirds of the patients in each cohort received the combination ribociclib treatment, while the remainder received letrozole alone. Single-cell RNA sequencing (scRNAseq) was performed on each serially collected sample of the tumors (detailed in (10)). In the discovery cohort, 35 patients provided high-quality biopsy samples yielding serial time-point scRNAseq (10X) data for analysis of cell type, phenotype, communication and composition (Figure 1). Of these patients, 23 received combination ribociclib (13 resistant and 10 sensitive tumors) and 12 received letrozole alone (5 resistant and 7 sensitive tumors). The validation cohort was sampled and processed following the same procedures and we additionally rescued some lower quality cells to retain a greater number of non-cancer cell types (especially immune cells) across samples. From the validation cohort biopsies, high-quality serial scRNAseq data were obtained for 27 patients, of which 16 received combination ribociclib (5 resistant and 11 sensitive tumors) and 11 received letrozole alone (7 resistant and 4 sensitive tumors). The discovery and validation cohorts were sequenced independently and in subsequent analyses (below) the validation cohort was used to replicate and verify key results detected in the discovery cohort.<br>Samples were excluded if insufficient RNA/DNA was obtained or due to poor quality.<br>Cell type annotation and verification<br>We obtained high quality transcriptional profiles for 424,581 single cells (41% discovery cohort, 59% validation cohort) with stringent quality controls ensuring high-coverage, low mitochondrial content, and high-confidence of doublet removal.
Replication	The findings presented in this paper were replicated in both the discovery and validation cohorts of the clinical trial.
Randomization	N/A
Blinding	N/A

Reporting for specific materials, systems and methods

We require information from authors about some types of materials, experimental systems and methods used in many studies. Here, indicate whether each material, system or method listed is relevant to your study. If you are not sure if a list item applies to your research, read the appropriate section before selecting a response.

Materials & experimental systems

n/a	Involved in the study	
☒	☐	Antibodies
☐	☒	Eukaryotic cell lines
☒	☐	Palaeontology and archaeology
☒	☐	Animals and other organisms
☐	☒	Clinical data
☒	☐	Dual use research of concern
☒	☐	Plants

Methods

n/a	Involved in the study	
☒	☐	ChIP-seq
☒	☐	Flow cytometry
☒	☐	MRI-based neuroimaging

Eukaryotic cell lines

Policy information about cell lines and Sex and Gender in Research

Cell line source(s)	CAMA1 cells (H. sapiens) American Type Culture Collection) (ATCC) identifier HTB-21+CAMA-1 (CRR)/CAMA-1_riboR_Cer2 Grolmusz et al, 2020; Emond et al, 2023)
Authentication	All cell lines were verified by STR profiling.
Mycoplasma contamination	Regular testing for mycoplasma contamination was conducted using the commercially available Myco Alert kit from Lonza.
Commonly misidentified lines (See ICLAC register)	NA

Clinical data

Policy information about clinical studies

All manuscripts should comply with the ICMJE guidelines for publication of clinical research and a completed CONSORT checklist must be included with all submissions.

Clinical trial registration	ClinicalTrials.gov Identifier: NCT02712723
Study protocol	Trial protocol published here : https://www.nature.com/articles/s43018-021-00215-7
Data collection	Patients were recruited from March 2016-August 2019. This study was led by Dr. Qamar Khan at the University of Kansas.
Outcomes	Resistance to therapy or growth during therapy were designated based on the imaging, pathological, and clinical measurements as described in the manuscript methods.

Plants

Seed stocks	NA
Novel plant genotypes	NA
Authentication	NA
